# Bread wheat satellitome: a complex scenario in a huge genome

**DOI:** 10.1007/s11103-023-01404-x

**Published:** 2024-01-30

**Authors:** Ana Gálvez-Galván, Manuel A. Garrido-Ramos, Pilar Prieto

**Affiliations:** 1 Plant Breeding Department, Institute for Sustainable Agriculture, Agencia Estatal Consejo Superior de Investigaciones Científicas (CSIC), Avda. Menéndez Pidal, Campus Alameda del Obispo S/N, 14004 Córdoba, Spain; 2 Departamento de Genética, Facultad de Ciencias, Universidad de Granada, Avda. Fuentenueva S/N, 18071 Granada, Spain

**Keywords:** Wheat, satDNA, Repetitive DNA, Subtelomeres, Telomeres, Chromosome recognition, Homologous pairing, Meiosis

## Abstract

In bread wheat (*Triticum aestivum* L.), chromosome associations during meiosis are extremely regulated and initiate at the telomeres and subtelomeres, which are enriched in satellite DNA (satDNA). We present the study and characterization of the bread wheat satellitome to shed light on the molecular organization of wheat subtelomeres. Our results revealed that the 2.53% of bread wheat genome is composed by satDNA and subtelomeres are particularly enriched in such DNA sequences. Thirty-four satellite DNA (21 for the first time in this work) have been identified, analyzed and cytogenetically validated. Many of the satDNAs were specifically found at particular subtelomeric chromosome regions revealing the asymmetry in subtelomere organisation among the wheat subgenomes, which might play a role in proper homologous recognition and pairing during meiosis. An integrated physical map of the wheat satellitome was also constructed. To the best of our knowledge, our results show that the combination of both cytogenetics and genome research allowed the first comprehensive analysis of the wheat satellitome, shedding light on the complex wheat genome organization, especially on the polymorphic nature of subtelomeres and their putative implication in chromosome recognition and pairing during meiosis.

**Supplementary Information:**

The online version contains supplementary material available at 10.1007/s11103-023-01404-x.

## Key message

Subtelomeres are enriched in particular satDNAs providing a specific pattern among chromosomes from each wheat subgenome, which might contribute to specificity in homologous chromosome recognition and pairing during meiosis in bread wheat.

**Supplementary Information:**

The online version contains supplementary material available at 10.1007/s11103-023-01404-x.

## Introduction

Bread wheat (*Triticum aestivum* L.) is one of the main crops in the world and a staple food in many countries. This species behaves as a diploid during meiosis despite its genome complexity (allohexaploid organism with 3 subgenomes from 3 different species). This means that homologous chromosomes (equivalent chromosomes from the same subgenome) recognize and associate in pairs in the presence of equivalent chromosomes from the other subgenomes (homoeologous chromosomes). Although the mechanisms involved in these processes have a strong influence on fertility and consequently, are critical in the framework of wheat breeding (Lukaszewski 2000; Shubing and Honggang 2005; Calderón et al. 2012; Rey et al. 2015), they remain still unknown. The distal region of the chromosomes, which includes telomeres and subtelomeres, are involved in the initial processes of recognition, pairing and recombination during meiosis in wheat (Calderón et al. 2014; Naranjo 2015). At the onset of meiosis, telomeres associate in a cluster that facilitates chromosome-end interactions for homologous recognition and pairing (Dawe 1998; Bass et al. 2000; Cowan et al. 2001; Harper et al. 2004; Naranjo 2014). This chromosome polarization with telomeres clustered in one side of the nucleus and centromeres in the opposite site occupying different spaces, allow telomeres and subtelomeres being in proximity and facilitate chromosomes interaction at the onset of meiosis (Rabl 1885; Anamthawat-Jónsson et al. 1990; Cowan et al. 2001; Oliver et al. 2013; Naranjo 2015; Doğan and Liu 2018).

Evidence supporting the implication of terminal region in homologous chromosomes recognition and pairing make strategic the study and characterization of telomeres and subtelomeres. Plant telomeres are highly conserved structures consisting in the consensus repetitive sequence 5’-TTTAGGG-3 described in *Arabidopsis thaliana* (Richards and Ausubel 1988). Subtelomeres are less evolutionary conserved than telomeres, gene-rich and contain highly repetitive DNA which might also include a variable number of telomere degenerate repeats (Linardopoulou et al. 2005; Louis and Vershinin 2005; Emden et al. 2019). Although there is not a clear functional or structural definition for subtelomeres, these regions are considered extremely relevant in recombination, genome stability, replication and other essential biological activities (Riethman 2008; Garrido-Ramos 2015, 2021; Aguilar and Prieto 2020). In the framework of meiosis, transcription and recombination have been also correlated to homologous chromosome recognition and pairing (Valenzuela et al. 2013; Hiraoka 2020; Aguilar and Prieto 2020). Additionally, subtelomeres can also contribute to protect genes located near the chromosome ends and to stabilise telomeric regions without telomeric repeats (Louis and Vershinin 2005). Despite the important roles that telomeres and subtelomeres seem to play, these chromosome regions are not well-characterized at the molecular level so far. For example, previous studies of all wheat chromosome ends revealed that only few chromosomes display the plant terminal telomeric repeat (5ť-TTTAGGG-3ť, or 3ť-AAATCCC-5ť on the complementary strand) in the available wheat sequencing database, developed by the International Wheat Genome Sequencing Consortium (IWGSC, Ref-Seq v1.1, https://www.wheatgenome.org) (Aguilar and Prieto 2020). Thus, a previous analysis of wheat subtelomeres was only possible in those chromosome displaying the telomeric repeat, finding a high polymorphism in the subtelomeric region between homoeologous chromosomes and supporting a role in chromosome specificity at the onset of meiosis (Aguilar and Prieto 2020). A deeper analysis of wheat subtelomeres should be done to shed light on other features such as repetitive DNA, which constitutes an important portion of these chromosome regions.

The number and complexity of repetitive DNA sequences varies among species, and include different types and families of transposable elements (TEs) and satellite DNAs (satDNAs), being more abundant in species with larger genomes (Kidwell 2002; Zhang et al. 2004; López-Flores and Garrido-Ramos 2012). In fact, repetitive DNA sequences account for more than 90% of wheat genome (Li et al. 2004), which is approximately 17 Gb (Appels et al. 2018). SatDNAs are noncoding repetitive DNA sequences organized in long tandem arrays which are concentrated in specific parts of the chromosomes such as centromeres, pericentromeric and subtelomeric regions, and interstitial regions of specific chromosomes, although short arrays and even single repeat units can be found throughout the whole genome of eukaryotic species (Garrido-Ramos 2015, 2017, 2021). SatDNA are organised into families according to sequence and repeat unit length, and each one is characterized by specific location, organization and repeat-copy number. The complete set of satDNA families of a genome is known as the satellitome (Ruiz-Ruano et al. 2016). Most satDNA families are species-specific or are shared by a reduced group of closely related species, constituting one of the most rapidly evolving fractions of the genome. However, satDNA families conserved in large groups of species for a long time have been described (Garrido-Ramos 2015, 2017, 2021). Related S genomes of diploid and polyploid *Triticum* and *Aegilops* species have been deeply studied using satDNAs among other DNA sequences in order to assess evolutionary changes between *Triticum* and *Aegilops* genomes (Ruban and Badaeva 2018). It is not clear whether satDNA sequences persist over time by selection or chance (Camacho et al. 2022), but some of them might play important roles in the eukaryotic genome having significant functions such as telomere stabilization or kinetochore nucleation (Garrido-Ramos 2015, 2017, 2021). In addition, the distribution of tandem repeated sequences seem to be important for chromosomes pairing and may, therefore, affect the meiotic stability of cereal hybrids (Metzlaff et al. 1986).

In this work we identified and characterized the bread wheat satellitome with the aim of going deeper into the knowledge of specific satDNAs that are concentrated on the terminal regions of chromosomes and might play particular functions on the processes of homologous chromosomes recognition and pairing during meiosis. Based on the existing information in the IWGSC RefSeq v2.1 genomic assembly (Zhu et al. 2021), RepeatExplorer2/TAREAN (Novák et al. 2010, 2013, 2020) and satMiner pipelines (Ruiz-Ruano et al. 2016), it has been possible to increase the likelihood of finding new rare satDNA families as previously done in species with a high C-value and/or a small amount of satDNA (Ruiz-Ruano et al. 2016, 2019). A total of 34 different satellite DNA families, corresponding to 2.53% of total bread wheat genome, have been identified, analyzed and cytogenetically validated. An integrated physical map of the wheat satelillome has been constructed. To the best of our knowledge, the combination of both, cytogenetic and genome research, have allowed the first comprehensive analysis of the wheat satellitome.

## Results

### High-throughput search for satDNAs

After five runs of filtering + RE performed on the bread wheat library, we found 34 satDNAs, defined by repeat-unit length and sequence (Table 1). Figure S1 shows the reconstruction of representative monomer sequences for each satDNA family. BLAST search found significant similarity between 13 satDNA families and other wheat satDNAs previously isolated by other procedures (Table S1). Therefore, the satDNA mining performed in this work revealed 21 new wheat satDNA families. BLAST search revealed the homology between some of these satDNAs and other satDNAs isolated from the genome of other Poaceae species (Table S1). Table 1 shows the main features of these satDNAs standing out the enormous diversity of the satellitome of *T. aestivum*. The length of the repeat units ranged between 44 (TaeSat13-44) and 2,619 bp (TaeSat03-2619), with three satDNAs being shorter than 100 bp and four being longer than 1,000 bp. Most of them (twenty-three) were A + T rich and ten were G + C rich. No correlation existed between satDNAs repeat-unit length and A + T content.

**Table 1:** Metrics of different parameters of satDNAs identified in bread wheat

Satellite Name	Length (nt)	Abundance (%)	Nº bp	Nº copies	Divergence (%)	AT content (%)	SF	DP	RPS	FISH (see Table 3)
TaeSat01-584	584	0,4729	80,175,466	137,287	0,0982	37,33		4	45,61	Dispersed
TaeSat02-118	118	0,3053	51,760,562	438,649	0,1253	48,31		12	43,09	Multiple locations
TaeSat03-2619	2619	0,2757	46,742,178	17,847	0,2257	63,38		24	41,02	Terminal
TaeSat04-337	337	0,2710	45,945,340	136,336	0,0813	65,28	SF-1	2	41,97	Multiple locations
TaeSat05-500	500	0,2204	37,366,616	74,733	0,2402	56,80	SF-2	21	29,58	Dispersed
TaeSat06-403	403	0,1815	30,771,510	76,356	0,2185	60,55		21	30,45	Dispersed
TaeSat07-343	343	0,1688	28,618,352	83,435	0,0667	59,18	SF-1	2	43,37	Multiple locations
TaeSat08-663	663	0,1066	18,072,964	27,259	0,0711	65,16	SF-3	4	55,98	Terminal
TaeSat09-335	335	0,1003	17,004,862	50,761	0,0972	36,12	SF-4	7	41,69	Multiple locations
TaeSat10-206	206	0,0892	15,122,968	73,412	0,0579	65,62	SF-1	2	53,46	Multiple locations
TaeSat11-506	506	0,0784	13,291,936	26,269	0,1244	62,85	SF-2	7	43,94	Dispersed
TaeSat12-369	369	0,0460	7,798,840	21,135	0,0508	60,16	SF-5	2	68,40	Terminal
TaeSat13-44	44	0,0348	5,899,992	134,091	0,0761	70,45		7	46,99	Multiple locations
TaeSat14-1463	1463	0,0261	4,424,994	3025	0,1825	59,13	SF-6	23	20,60	(peri)centromeric
TaeSat15-620	620	0,0260	4,408,040	7110	0,2466	62,58		29	26,89	Multiple locations
TaeSat16-567	567	0,0199	3,373,846	5950	0,1161	53,62	SF-5	4	35,83	(peri)centromeric
TaeSat17-323	323	0,0143	2,424,422	7506	0,0890	62,23		7	54,80	Terminal
TaeSat18-733	733	0,0121	2,051,434	2799	0,0792	66,58		4	48,83	Multiple locations
TaeSat19-653	653	0,0111	1,881,894	2882	0,1770	54,52	SF-3	2	40,24	Multiple locations
TaeSat20-322	322	0,0096	1,627,584	5055	0,1574	39,75	SF-4	4	25,59	Terminal
TaeSat21-1590	1590	0,0091	1,542,814	970	0,0659	35,60		2	55,99	Dispersed
TaeSat22-320	320	0,0079	1,339,366	4186	0,0879	48,12	SF-6	7	42,83	Terminal
TaeSat23-319	319	0,0075	1,271,550	3986	0,1082	42,01	SF-6	4	39,36	Terminal
TaeSat24-338	338	0,0069	1,169,826	3461	0,1338	40,83		5	26,83	Multiple locations
TaeSat25-318	318	0,0048	813,792	2559	0,1595	45,60	SF-6	12	20,87	Multiple locations
TaeSat26-210	210	0,0045	762,930	3633	0,0324	59,52		0	80,40	Terminal
TaeSat27-72	72	0,0044	745,976	10,361	0,0829	50,00		4	43,68	Terminal
TaeSat28-543	543	0,0040	678,160	1249	0,1078	55,43		5	42,92	(peri)centromeric
TaeSat29-319	319	0,0035	593,390	1860	0,1039	47,02	SF-6	2	37,69	Multiple locations
TaeSat30-1389	1389	0,0032	542,528	391	0,1373	63,43		2	31,23	Multiple locations
TaeSat31-889	889	0,0029	491,666	553	0,0280	59,39		2	91,49	(peri)centromeric
TaeSat32-528	528	0,0019	322,126	610	0,0237	56,63		0	90,80	Terminal
TaeSat33-54	54	0,0006	101,724	1884	0,2204	72,22		19	42,69	Multiple locations
TaeSat34-175	175	0,0004	67,816	388	0,1621	70,69		18	26,46	Multiple locations
		2,5316	429,207,464	1,367,987						

Length (nt), abundance (% of the genome), nº bp (bases pair), nº copies, divergence (%), A + T content (%), superfamilies (SF), DIVPEAK (DP), relative peak size (RPS) and FISH. FISH: dispersed (satDNAs with scattered signal along the whole chromosome); (peri)centromeric (satDNAs with positive signal around the centromere of the chromosomes); terminal (satDNAs with signal located in the terminal regions of the chromosomes (subtelomeres); multiple locations (satDNAs with undefined signal in a specific chromosome region (terminal, centromeric, interstitial)). See Table 2 for further information about FISH

### SatDNA abundance and divergence

Collectively, all 34 satDNAs represent approximately 2.53% of the genome of *T. aestivum* (Table 1). That is, assuming a genome size of this species of about 16,954 Mbp (Bennett and Smith 1976), this percentage represented about 430 Mbp. Individual satDNA family abundance ranged between 0.0004% and 0.47% of the genome. 11 families were among the most abundant in the genome (> 0.05% of the genome), while 16 families were scarcely represented (< 0.01% of the genome). TaeSat02-118 satellite family stands out with more than 400,000 copies, being the second most abundant satDNA, while exist three other satDNAs with more than 100,000 copies (TaeSat01-584, TaeSat04-337 and TaeSat13-44; this latter being only 0.035% of the genome since it is composed of short repeats of 44 bp). TaeSat34-175 is the satellite with the smallest number of copies (388 repeats), being also the least abundant (0.0004% of the genome). Eight out of 13 previously published satDNAs identified by other methods are among the most abundant satellites found in this study, something that denotes a relationship between abundance and affordability for their isolation by conventional methods. However, among the most abundant ones, there are several satDNAs identified in this paper for the first time (TaeSat01-584, TaeSat06-403 and TaeSat08-663).

Intra-familiar divergence ranged between 2.37 and 25.31%. No significant correlation was found between satDNA abundance and divergence. Likewise, abundance and divergence failed to show significant correlation with unit length or A + T content. However, there is a positive correlation between intrafamiliar divergence and DIVPEAK (DP) and a negative correlation between intrafamiliar divergence and RPS (r = 0.771; p < 0.01). Figure S2 displays repeat landscape plots representing, for each satDNA, abundance (Y axis) and divergence (X axis) with respect to the consensus sequence built for each satDNA repeat unit. These profiles result highly informative on the age of satDNA variants within a same family since peaks at lower divergence values are the product of more recent amplification and homogenization events, whereas those at higher divergence values are older variants that have accumulated many mutations. There are several satDNAs sharing similar DP values but somewhat differing in their divergence values, and vice versa, which suggests different turnover rates for different satDNA families. We cannot have an estimation of the substitution rates for *T. aestivum* satDNAs. However, using any published value from any other satellites, even the highest value, the time for all DP, i.e. the time for the last amplification event, predates the origin of this allohexaploid species, ~ 8000 years ago, and the domestication of *T. turgidum* (Emmer allotetraploid wheat), ~ 10,000 years ago. Therefore, the last amplification event for most of these satDNAs must have occurred in the diploid parental species. Notwithstanding, satellites TaeSat15-620, TaeSat26-210, TaeSat29-319, TaeSat30-1389, TaeSat31-889 and TaeSat32-528 are now apparently in a process of expansion as can be seen in the RLs plots.

### SatDNA internal organization

The consensus sequence of every satDNA analyzed in this paper shows internal direct subrepeats indicative of past events of repeat length amplifications. Interestingly, there exist correlation between repeat length and number of subrepeats (r = 0.877; p < 0.01), which supports the origin of larger repeats units from shorter ones. In fact, more than 50% of the current length of the repetitive unit of most satDNAs (28 in total) is explained by these internal subrepeats (Table S2). TaeSat15-620 stands out as it is composed of 4 subrepeats of 155 bp, which demonstrates that this might be the original repeat length of this satDNA.

We have also analyzed the consensus sequence of each satDNA for the presence of putative inverted subrepeats. All families are composed of numerous putative inverted subrepeats. Many of them are short, ranging between 5 and 9 bp (Table S3). However, there are an important number of dyad symmetries longer than 10 bp in most satellites (Table S3). There is no correlation between repeat length and maximum length of the inverted repeats but repeat length and number of inverted repeats correlate (r = 0.930; p < 0.01). We also checked all satDNAs for nucleic acid folding prediction, estimating Gibbs free energy (dG) of the predicted secondary structures (Table S3). There is a correlation between the dG value and both the length of the satellite (r = 0.864; p < 0.01) and the number of inverted subrepeats both greater and lesser than 10 bp (r = 0.933; p < 0.01). It is noteworthy that most of the satellites show very high free energy values with 27 having dG values < -30. All these 27 satDNAs have a repeat unit length > 300 bp. Centromeric satellites show high dG values but they are not the highest ones. The complex structure observed for satDNAs analyzed in this paper is translated in that all of them show a high probability to acquire particular stable non-B-form conformations such as stem-loops or cruciforms (Figure S3).

We have found that most of the satDNA families show a certain propensity to acquire stable curvatures (Table S4). However, the magnitude of these peaks for most satDNAs (> 14 degrees/10.5 bp helical turn) was low and, in most cases, even far from the values calculated for other highly curved motifs. Notably, TaeSat23-319 has a prominent peak of 15 degrees/10.5 bp helical turn and TaeSat08-663, TaeSat18-733 and TaeSat28-543 have a prominent peak of about 16 degrees/10.5 bp helical turn. Interestingly, TaeSat28-543 is centromeric (see below) but not the other, and conversely TaeSat14-1463, TaeSat16-567 and TaeSat31-889 (other centromeric satDNAs) show curvature peaks lower than 12.2 degrees/10.5 bp helical turn.

### Similarities between satDNA families

We have detected 6 superfamilies (SF), i.e., 6 groups composed of homologous satDNAs families that probably derived from a common ancestor satDNA (Table 1). Figures S3-S8 show the relationships between members of each superfamily. Superfamily 1 (SF-1) is composed by the families TaeSat04-337, TaeSat07-343 and TaeSat10-206 (Figure S3). TaeSat04-337 and TaeSat07-343 share 82.2% of similarity while they share 98% and 82.5%, respectively with TaeSat10-206. Both TaeSat04-337 and TaeSat07-343 have a duplicated AAATGATGAT sequence flanking the inserted region with respect to TaeSat10-206, which lacks such duplication. SF-2 is composed by TaeSat05-500 and TaeSat11-506 that are 68% similar along a contiguous fragment of about 400 bp but they are totally different in the first 108 bp (Figure S4). SF-3 is composed of TaeSat08-663 and TaeSat19-653 that share 70% identity in a fragment of 486 bp (Figure S5). SF-4 is composed of TaeSat09-335 and TaeSat20-322 that are 70% similar (Figure S6). SF-5 is composed of TaeSat12-369 and TaeSat16-567, which only share 51 bp (88% identity). SF-6 is a complex superfamily composed of TaeSat22-320, TaeSat23-319, TaeSat25-318, TaeSat29-319 and TaeSat14-1463. Although the length of the repeat unit is very similar in four of them, their sequence is sufficiently distinctive (Figure S7). Indeed, the divergence between these satDNAs is quite important in several parts of the monomer length to such an extent that the four satDNAs only retain identity (68–75%, depending on the comparisons) in part of the sequence (between 25 and 82%, depending on the comparisons). Figures S7 and S8 show the patterns of similarities between these four sequences (TaeSat22-320, TaeSat23-319, TaeSat25-318 and TaeSat29-319). Furthermore, TaeSat14-1463 only retained between 82 and 218 bp (depending on the compared satDNA) sharing identity (73–79%) with the other four satDNA families.

### Chromosomal location of the wheat satDNAs

**Fig. 1:**
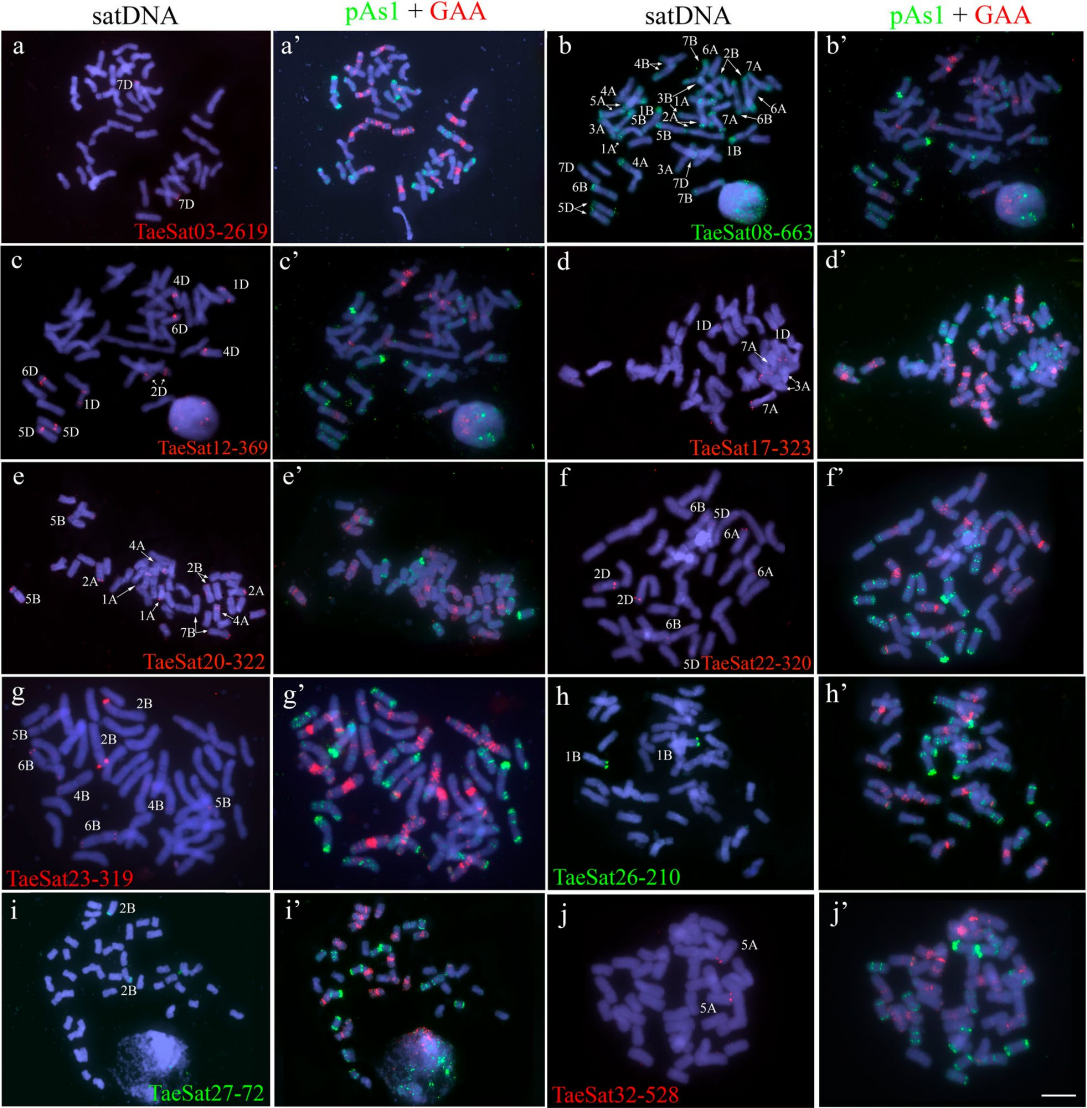
Cytogenetic visualization by fluorescent in situ hybridization of distal/subtelomeric satDNAs in metaphase chromosomes from *Triticum aestivum* cv. Chinese Spring. DNA was counterstained with DAPI (blue). SatDNAs were indistinctly labelled in red or green. Chromosome identification and orientation were confirmed by reprobing of the chromosome spreads with the pAs1 (green) and GAA (red) probes (panels **a’**–**j’**). **a** TaeSat03-2619, **b** TaeSat08-663, **c** TaeSat12-369, **d** TaeSat17-323, **e** TaeSat20-322, **f** TaeSat22-320, **g** TaeSat23-319, **h** TaeSat26-210, **i** TaeSat27-72 and **j** TaeSat32-528. Scale bar = 10 µm for all panels except for **g** and **f** where represents 7 µm

**Fig. 2:**
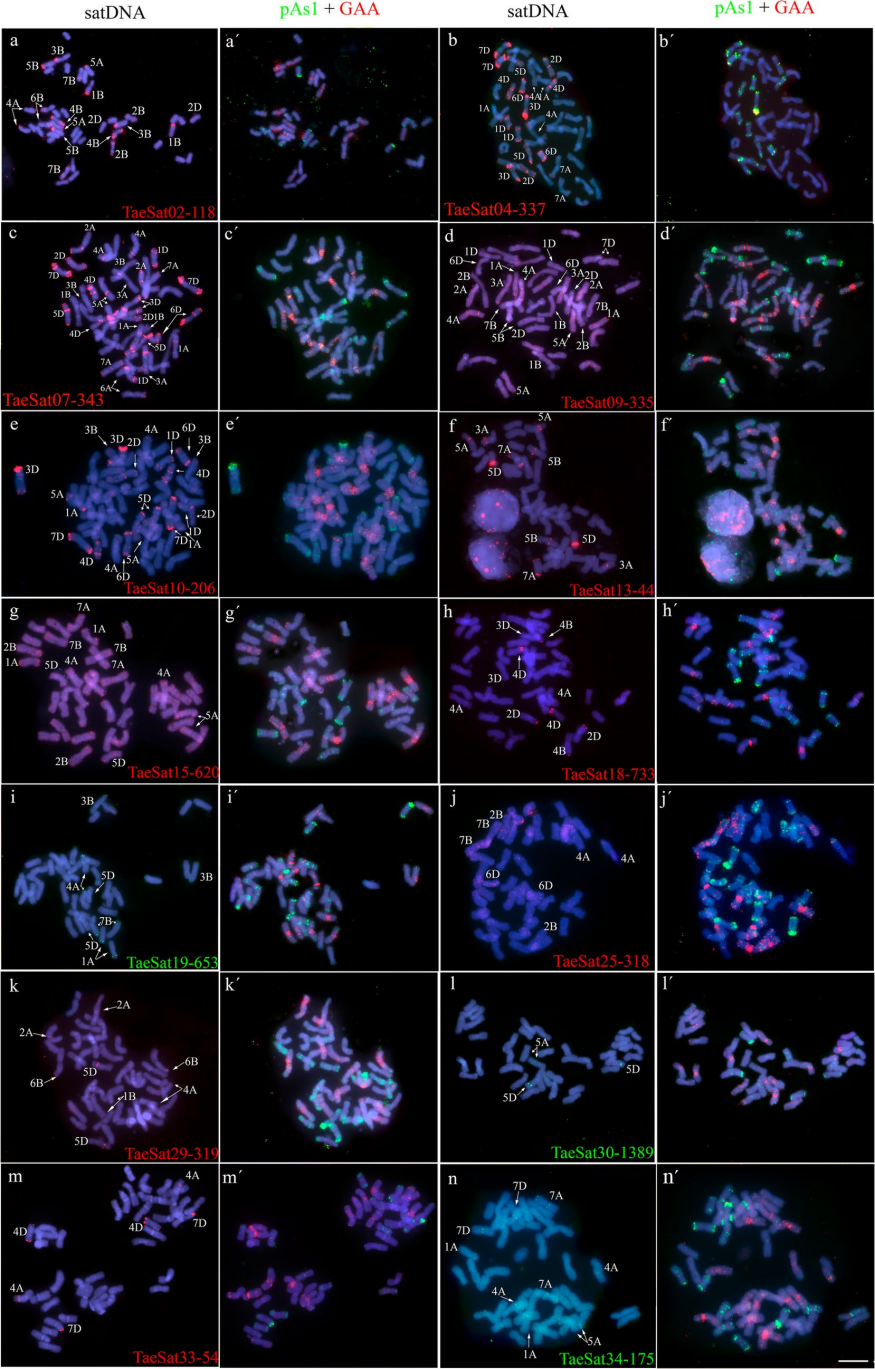
Cytogenetic visualization by FISH of satDNAs with a multiple location pattern (terminal/subtelomeric and some centromeric and/or interstitial signals) in metaphase chromosomes from *Triticum aestivum* cv. Chinese Spring. DNA was counterstained with DAPI (blue). SatDNAs were indistinctly labelled in red or green. Chromosome identification and orientation were confirmed by reprobing of the chromosome spreads with the pAs1 (green) and GAA (red) probes (panels **a’**–**n’**). **a** TaeSat02-118, **b** TaeSat04-337, **c** TaeSat07-343, **d** TaeSat09-335, **e** TaeSat10-206, **f** TaeSat13-44, **g** TaeSat15-620, **h** TaeSat18-733, **i** TaeSat19-653, **j** TaeSat25-318, **k** TaeSat29-319, **l** TaeSat30-1389, **m** TaeSat33-54 and **n** TaeSat34-175. Scale bar = 10 µm

**Fig. 3:**
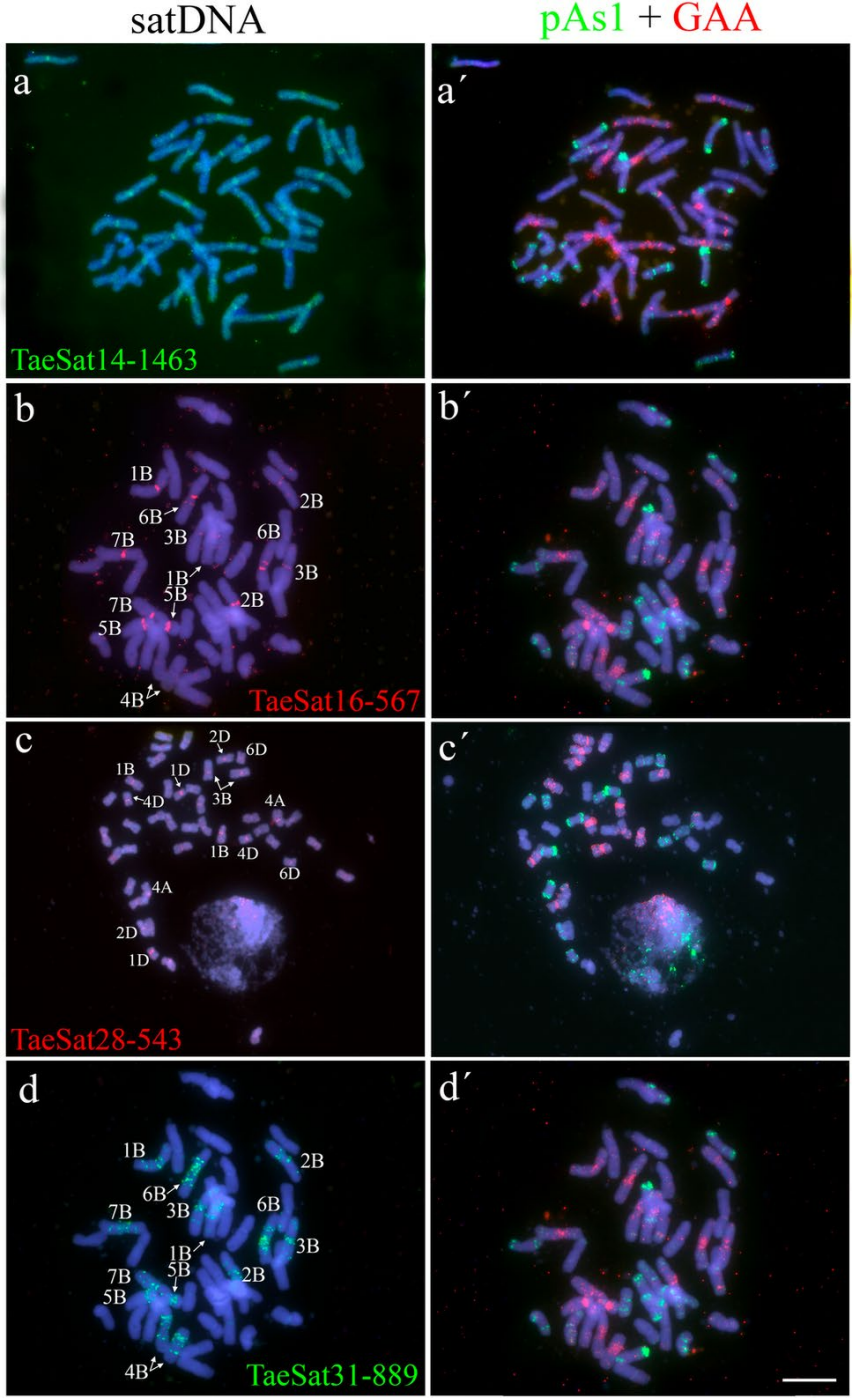
Cytogenetic visualization by FISH of centromeric satDNAs in metaphase chromosomes from *Triticum aestivum* cv. Chinese Spring. DNA was counterstained with DAPI (blue). SatDNAs were indistinctly labelled in red or green. Chromosome identification and orientation were confirmed by reprobing of the chromosome spreads with the pAs1 (green) and GAA (red) probes (panels **a’**–**d’**). **a** TaeSat14-1463, **b** TaeSat16-567, **c** TaeSat28-543 and **d** TaeSat31-889. Scale bar = 10 µm in all panels except in c) where represents 15 µm.

**Fig. 4:**
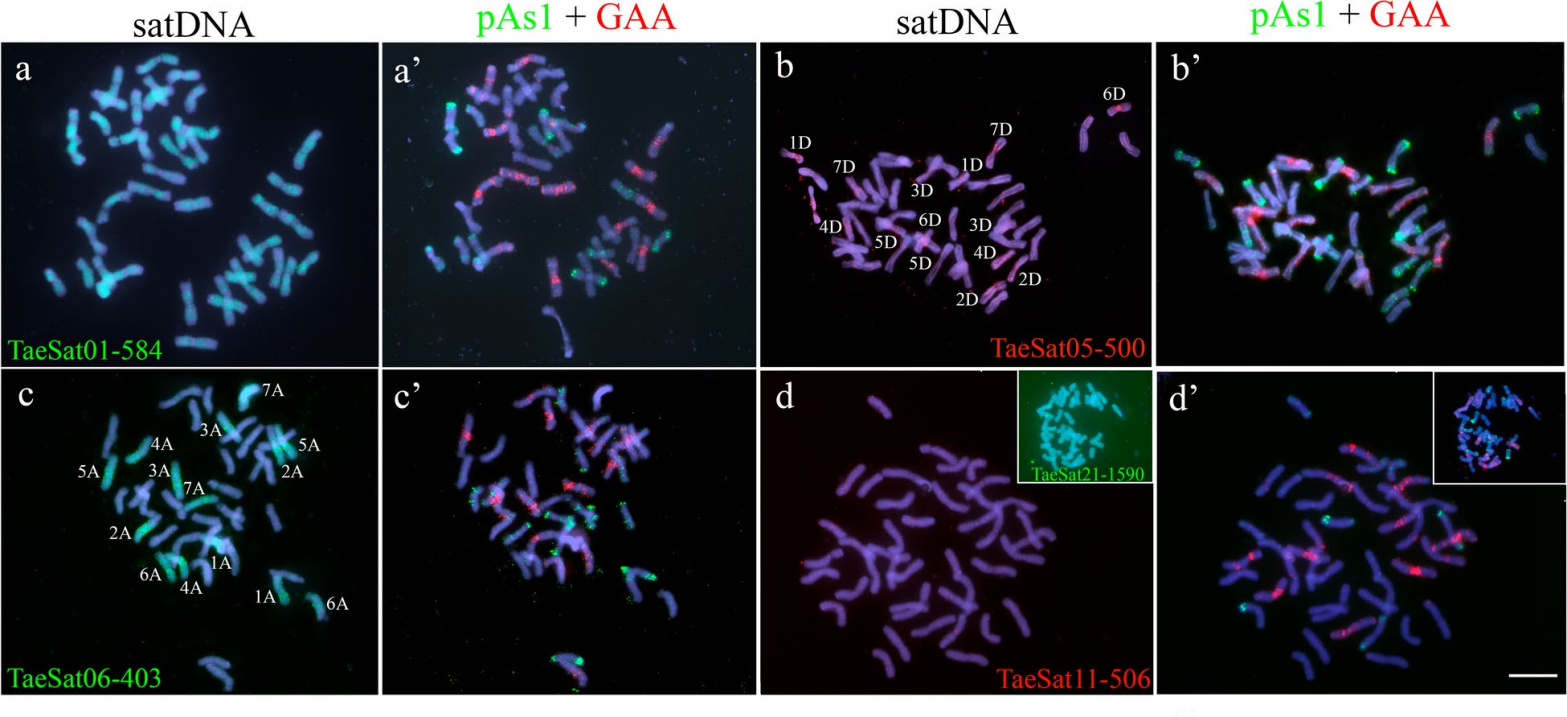
Cytogenetic visualization by FISH of satDNAs with a non-banded pattern (dispersed) in metaphase chromosomes from *Triticum aestivum* cv. Chinese Spring. DNA was counterstained with DAPI (blue). SatDNAs are indistinctly labelled in red or green. Chromosome identification and orientation were confirmed by reprobing of the chromosome spreads with the pAs1 (green) and GAA (red) probes (panels a’-d’). TaeSat01-584, with a dispersed patter in all chromosomes from the three wheat subgenomes. **b** TaeSat05-500 is mainly visualized in chromosomes from D subgenome with stronger signals in centromeres and pericentromeric regions. **c** TaeSat06-403 has a dispersed patter to all chromosomes from A subgenome. **d** TaeSat11-506 (and TaeSat21-1590 in the inset) with a dispersed pattern in all chromosomes from the three wheat subgenomes. Scale bar = 10 µm

**Fig. 5:**
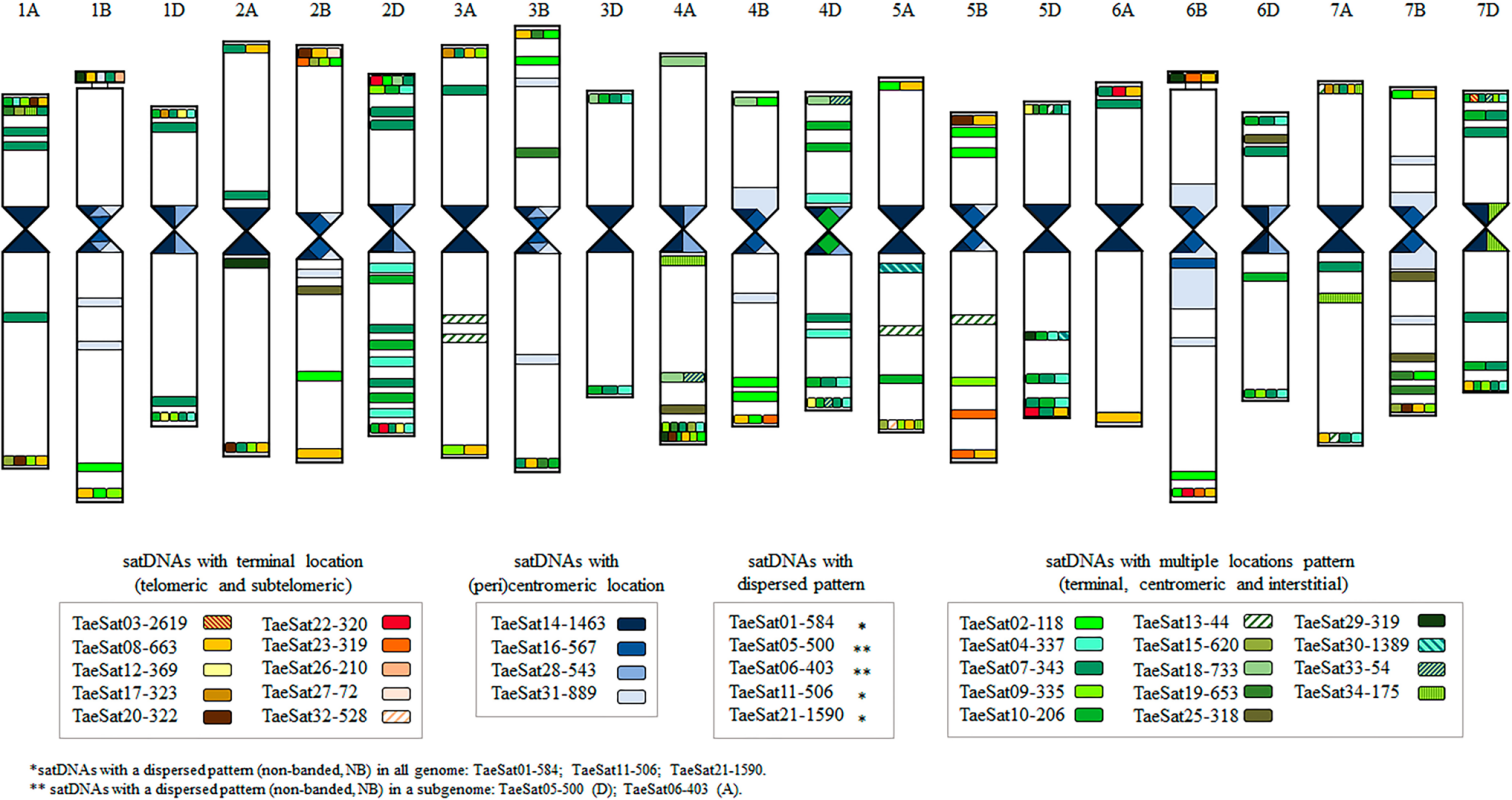
Idiogram of *Triticum aestivum* cv. Chinese Spring chromosomes representing satellites DNA location identified in this work by fluorescent in situ hybridization (FISH). SatDNAs were grouped according to their location patterns: satDNAs with distal chromosome location (telomeric and subtelomeric) in a brown color scale; satDNAs with (peri)centromeric location; Green scale: satDNAs with multiple locations pattern (terminal, centromeric and interstitial). (*) Satellites DNA with a dispersed pattern in all genome: TaeSat01-584, TaeSat11-506 and TaeSat21-1590; (**) Satellites DNA with a dispersed pattern in a subgenome: TaeSat05-500 (D subgenome); TaeSat06-403 (A subgenome) are not represented by any colour on the ideogram

We were able to obtain PCR probes for FISH of all satDNAs except TaeSat24-338. Most of the satDNAs identified in this work displayed FISH signals in the distal/subtelomeric chromosome regions although some of them also included some others in interstitial and/or (peri)centromeric positions simultaneously in the wheat chromosomes. Only four satDNAs were exclusively visualized at the (peri)centromeric chromosome regions. That is, depending on the FISH cytogenetic pattern, the 33 satDNAs, were organised in four different groups: satDNAs clustered exclusively at terminal subtelomeric chromosome regions (Fig. 1); satDNAs clustered at the distal (subtelomeric) chromosome regions but also including simultaneously some other FISH signals in (peri)centromeric and/or interstitial regions (Fig. 2); satDNAs clustered at the (peri)centromeric region (Fig. 3); and satDNAs that had a dispersed non-clustered pattern either in all wheat chromosomes or in the chromosomes from one specific subgenome, but also included FISH signals at the subtelomeric regions (Fig. 4). The distribution pattern of all satDNAs was also schematically represented in Fig. 5 and Supplementary Figure S10 to facilitate their simultaneous visualization.

The group of the exclusive subtelomeric satDNAs included 10 satDNAs (Fig. 1; Table 2). Four of these satDNAs (TaeSat03-2619, TaeSat26-210, TaeSat27-72 and TaeSat32-528) showed only a single band in just one pair of homologous chromosomes (7DS, 1B-organizer, 2BS and 5AL; respectively). TaeSat12-369 and TaeSat23-319 signals were specific to the subtelomeric regions of the chromosomes from D and B subgenome, respectively (1DS/1DL, 2DL, 4DL, 5DS and 6DS; 2BS, 4BL, 5BL and 6B-organizer/6BL chromosomes). TaeSat08-663 had positive signals at both ends of all chromosomes from A and B subgenomes, and on the long arm of 4A, 4B, 5D and 7D chromosomes. Finally, TaeSat17-323, TaeSat20-322 and TaeSat22-320 had positive FISH signals in some chromosome pairs: 1DS, 3AS and 7AS; 1AS-1AL, 2AL, 2BS, 4AL, 5BS and 7BL; and 2DS-2DL, 5DL, 6AS and 6BL, respectively.

**Table 2:**
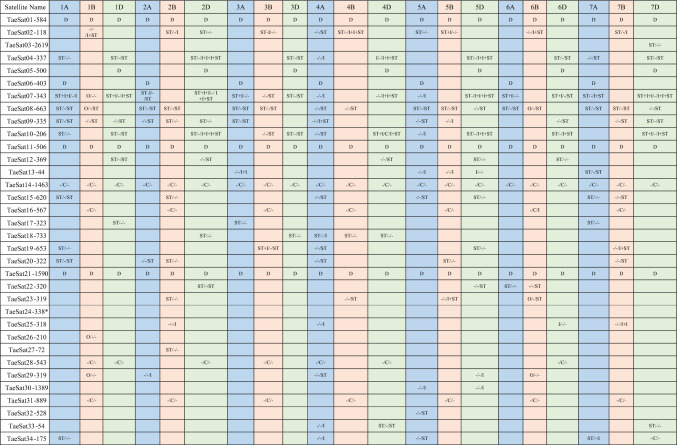
Summary of the fluorescence in situ hybridization (FISH) patterns for the different bread wheat (*Triticum aestivum* cv. Chinese Spring) satellite DNA families identified in this work

The different lines collect the result to individual satDNA. Each column collects the information to individual chromosome. The different subgenomes are differentiated by colour (A = blue; B = pink; D = green). Each cell represents: Short arm/ Centromere/ Long arm; ST = subtelomere; I = intersticial; C = (peri)centromeric; D = dispersed; O = Organizer

A second group is composed of another 14 subtelomeric satDNAs, although all of these satDNAs also showed some additional strong FISH signals in the (peri)centromeric, and/or interstitial chromosomal regions, including the nucleolus organizer (Fig. 2; Table 2). In addition to the banded pattern, some of these satDNAs (TaeSat09-335 and TaeSat15-620, for example) showed a dotted pattern indicating an additional dispersed distribution throughout the genome.

Four satDNAs (TaeSat14-1463, TaeSat16-567, TaeSat28-543 and TaeSat31-889) revealed an exclusive (peri)centromeric pattern on the wheat chromosomes, being two of them (TaeSat16-567 and TaeSat31-889) specific of the B subgenome (Fig. 3; Table 2). In detail, TaeSat14-1463 was mainly visualised in the (peri)centromeres of all chromosomes, although dispersed signals all over the genome were also observed. TaeSat16-567 signals were detected in all (peri)centromeres of the B subgenome, with weaker signals in centromeres of 2B, 3B and 4B chromosomes. TaeSat28-543 showed FISH signals in all wheat centromeres although strongest signals can be observed in the centromeres of 1B, 1D, 2D, 3B, 4A, 4D and 6D chromosomes (Fig. 3c). TaeSat31-889 was only visualized in all (peri)centromeric regions of the chromosomes from the B subgenome, although it also extends pericentromerally to interstitial locations.

Finally, there are 5 satDNAs having a dispersed chromosome pattern but also including FISH signals in the subtelomeric regions (Fig. 4; Table 2). In detail, some satDNAs displayed a dispersed pattern throughout all wheat chromosomes (TaeSat01-584, TaeSat11-506 and TaeSat21-1590) and another two satDNAs revealed strongest signals only in the chromosomes of one subgenome (TaeSat05-500 and TaeSat06-403 in chromosomes from D and A subgenomes, respectively). It is noteworthy that although these satDNAs have a dispersed chromosome pattern including the subtelomeric regions, in some cases, strongest FISH signals can be observed in some certain chromosome regions, indicating possibly a higher accumulation of repeats in those chromosome regions (for example TaeSat01-584 and TaeSat06-403).

### BLAST search of satDNAs to the genome of *T. aestivum*

Tracing the genome assembly of this species with the satDNAs identified in this work revealed three relevant aspects (Table S5). Firstly, many of the loci that appear as conspicuous FISH bands of a given satDNA corresponded to regions where the assembly of the repetitive units of the satDNA in question has collapsed into only a few of these tandem repeats or even directly generated a gap. For example, although TaeSat31-889 was visualized by FISH in the peri(centromeric) region of all chromosomes from the B subgenome, BLAST only identified a high number of hits, some of them consisting in large tandems of repeats, in chromosome 1B, in addition to one or two hits in chromosomes 5B, 6B and 7B, but none in the rest of B chromosomes (2B, 3B and 4B) (compare Tables S5 and 2).

Secondly, a major concentration of the repeated sequences of these satDNAs analyzed in this paper are delocalized appearing in the maze of discarded sequences that form the so-called unplaced chromosome of the genome assembly, revealing again the deficiencies in the available assembly of wheat genome.

Thirdly, all satDNAs shared that, in addition to the major loci detected by FISH, they had copies of the repeat unit scattered throughout the genome, either individually isolated or consisting of very short tandem arrays undetectable by FISH (see for example Table 2 and Table S5 for TaeSat04-337 or TaeSat10-206). In this context, it is noteworthy that several satDNAs showed a FISH signal only in a chromosome pair (for example, TaeSat03-2619 and TaeSat27-72) but also showed single repeat units scattered throughout the whole genome (not detectable by FISH). Curiously, these scattered single repeats were shorter than 2619 bp for TaeSat03-2619 (hits consisting in tandems of 2–3 complete repeat units of this satDNA were only located in the unplaced chromosome). In contrast, beside single hits identified on 4B and 4D chromosome pairs non-detectable by FISH, another 439 hits of TaeSat32-528, grouped in large tandems, were found in 5AL chromosome, which did match with FISH results. Similarly, FISH signals for TaeSat30-1389 were detected in 5AL and 5DL homoeologous chromosomes, but BLAST hits were found in chromosomes 5A (125 hits), 5B (18 hits), 5D (164 hits), 7A (2 hits) and 7D (2 hits), as well as some in the unplaced chromosome. As expected, the highest number of hits corresponded to those with FISH signal (5A and 5D), and only the full length of the satDNA (1389 bp) was found on chromosome 5D.

In addition to these general conclusions, it is worth mentioning the BLAST results for those satDNAs displaying a dispersed FISH pattern throughout the whole genome (for example TaeSat01-584) or just in one subgenome (TaeSat05-500 and TaeSat06-403 in D and A subgenomes, respectively). Depending of the satDNA, BLAST search revealed hundreds/thousands of hits per chromosome composed by one, two or few tandem repeats, coinciding with the scattered FISH pattern. As mentioned above, some of these satDNAs displayed denser FISH signals that can be visualized as solid blocks in specific chromosome regions corresponding with a BLAST accumulation of many repeats close together in these particular regions of some chromosomes. That is the case for the hits that TaeSat01-584 displayed in chromosome 3D (analyzed in a separate sheet in Table S5), in which arrays longer than 2 or 3 tandem repeats have not been found. Curiously, TaeSat05-500, which appears as a D-specific satDNA by FISH, was also detected in the other two subgenomes by BLAST analysis, as it also happens to TaeSat06-403, which was visualized by FISH throughout the A subgenome although BLAST analysis of the bread wheat genome reveals its presence throughout all three subgenomes. That is, although thousands of hits per chromosome were identified in A subgenome only few hundred in distant positions in B and D subgenomes.

Finally, it is remarkable that BLAST analysis for TaeSat24-338 revealed hits consisting in tandems of 2–3 copies detected on all wheat chromosomes. For unexpected reasons, it was not possible to amplify this sequence by PCR and consequently, design a probe for FISH visualization.

On the other hand, we checked the presence of all these satDNAs in subtelomeric regions. For this, we studied not only the IWGSC assembly but also another two assemblies available in the databases, such as the assembly provided by the John Hopkins University for the same Chinese Spring cultivar (PRJNA392179, Refseq v2.1, https://www.ncbi.nlm.nih.gov/datasets/genome/GCF_018294505.1/) as well as another assembly for the hexaploid wheat variety Fielder (PRJEB44721, https://www.ncbi.nlm.nih.gov/bioproject/PRJEB44721/), provided by Kazusa DNA Research Institute, Kisarazu, Japan. After downloading the assemblies of each chromosome individually, we identified those chromosomes that included the telomeric sequence [(TTTAGGG)n] resulting in only a few chromosomes (Table S6). Nevertheless, we selected the 500 Kb from each end of every chromosome and checked them for the presence of satDNAs. Our analysis revealed that many of the satDNAs analyzed (18 in total) have between one and several repeats in the chromosome ends (Table S6), but remarkably, there are few coincidences between what is observed in the assembly and what is detected by FISH, again highlighting the deficiency in the assembly of satDNAs.

### Relationship of bread wheat satDNAs with transposable elements

Analysis using RepeatMasker revealed that more than half of the sequences identified in this article as satDNAs (18 of 34) show homology with transposable elements (Table 3). Two relevant facts stand out in this analysis. The first is that not only the sequences that have a dispersed location throughout the genome showed homology with TEs. A total of 12 satDNAs showing conspicuous FISH bands in the classical mode (in addition to a multitude of repetitive unit locations scattered throughout the genome), are related to TEs. Specifically, the 16% of the sequence of TaeSat03-2619 is homologous to LTR/Gypsy retrotransposons and the 90% of the sequence of TaeSat15-620 is homologous to LTR/copia retrotransposons. Furthermore, 8 satDNAs (TaeSat04-337, TaeSat07-343, TaeSat08-663, TaeSat09-335, TaeSat14-1463, TaeSat19-653, TaeSat20-322 and TaeSat27-72) are homologous to DNA/CMC-EnSpm transposons. In most of them, the complete sequence is homologous to this type of elements. Therefore, these data reveal an origin of these satDNAs from TEs.

**Table 3:** Homology of satDNAs with transposable elements

		Percentage				Position in query						
Query sequence	SW score	Div	Del	Ins	Begin		End	Strand	Matching repeat	Repeat class/family	Coverage (%)	Genome distribution
TaeSat01-584	415	18.4	5.4	16.7	102		396	C	EnSpm-1_SIt	DNA/EnSpm/CACTA	50.3	Dispersed
TaeSat03-2619	1146	23.1	5.3	1.8	57		382	C	SABRINA3_TM-int	LTR/Gypsy	12.4	Terminal
	346	21.5	7.8	0.0	2087		2193	+	SABRINA3_TM-LTR	LTR/Gypsy	4.0	
TaeSat04-337	2905	2.7	0.0	0.0	1		336	+	EnSpm-N1_AT	DNA/CMC-EnSpm	100.0	Multiple locations
TaeSat05-500	226	28.0	4.9	4.9	50		193	+	EnSpm-4_SIt	DNA/EnSpm/CACTA	28.6	Dispersed
TaeSat06-403	1380	21.4	2.4	2.4	30		402	+	ERIKA1_TM-LTR	LTR/Gypsy	92.3	Dispersed
TaeSat07-343	2663	8.4	0.0	0.0	1		343	+	EnSpm-2_TD	DNA/CMC-EnSpm	100.0	Multiple locations
TaeSat08-663	5387	5.0	0.0	0.0	1		663	C	EnSpm-N1_TA	DNA/CMC-EnSpm	100.0	Terminal
TaeSat09-335	761	18.6	7.3	8.1	3		335	+	EnSpm1_TD	DNA/CMC-EnSpm	100.0	Multiple locations
TaeSat11-506	272	30.6	5.9	6.5	169		493	+	EnSpm-4_SIt	DNA/CMC-EnSpm	64.0	Dispersed
TaeSat14-1463	639	16.7	2.3	15.3	1147		1400	C	EnSpm-4_HV	DNA/CMC-EnSpm	17.3	(peri)centromeric
TaeSat15-620	324	22.7	2.2	2.2	4		93	C	EnSpm-N1_TA	DNA/CMC-EnSpm	14.3	Multiple locations
	722	24.4	4.1	4.4	60		618	C	Copia-6_TA-LTR	LTR/Copia	90.0	
TaeSat18-733	234	17.9	10.4	3.0	319		386	C	MuDR-N106B_OS	DNA/MuDR	9.1	Multiple locations
TaeSat19-653	1374	29.0	2.3	0.2	166		628	C	EnSpm-N1_TA	DNA/CMC-EnSpm	70.8	Multiple locations
TaeSat20-322	2663	2.8	0.6	0.3	1		322	+	EnSpm1_TD	DNA/CMC-EnSpm	100.0	Terminal
TaeSat21-1590	6231	9.1	6.8	3.0	1		1039	+	EWAY1_TM-int	LTR/Gypsy	65.3	Dispersed
	1989	3.7	6.5	0.4	1040		1285	+	EWAY1_TM-LTR	LTR/Gypsy	15.5	
	1798	4.3	0.0	8.8	1286		1590	+	EWAY1_TM-int	LTR/Gypsy	19.2	
TaeSat27-72	245	16.7	0.0	0.0	3		44	C	EnSpm3_TM	DNA/CMC-EnSpm	56.9	Terminal
TaeSat30-1389	232	15.2	13.9	2.5	829		908	+	MuDR-22_VV	DNA/MuDR	5.7	Multiple locations
TaeSat34-175	270	23.8	5.0	2.5	6		86	+	MuDR-26_ZM	DNA/MuDR	45.7	Multiple locations

Different columns indicate the following data: Smith-Waterman score of the match, complexity adjusted (SW score); percentage of substitutions in matching region compared to the consensus (Div); percentage of bases opposite a gap in the query sequence (deleted bp); percentage of bases opposite a gap in the repeat consensus (inserted bp); position in query (begin-end); strand (transcript strand ( +); complementary strand (C); matching repeat; repeat class/family; percentage of matching satDNA sequence and genome distribution (FISH signal)

Secondly, part of the sequence (between 17 and 100%, depending on the satDNA) in those satDNAs that have a scattered distribution throughout the genome (Fig. 4), revealed homology with LTR/Gypsy (TaeSat06-403 and TaeSat21-1590) or with DNA/CMC-EnSpm/CACTA elements (TaeSat01-584, TaeSat05-500 and TaeSat11-506). Therefore, part of these elements might come from TEs or alternatively, some of these elements are part of a TE.

## Discussion

### Completeness of bread wheat satellitome

Eukaryotic genome assembly algorithms have encountered a major difficulty in identifying and adequately characterising satDNA families included in a species satellitome due to their high degree of repetitiveness, so that satDNAs typically are underrepresented in genome assemblies (Henikoff 2002; Kapustová et al. 2019; Peona et al. 2021). This has resulted in genome assembly collapsing at the level of tandem repeat-rich regions of a genome, appearing as short loci with very few repeats or directly as gaps in which information has been completely lost (Chaisson et al. 2015; Kapustová et al. 2019). In fact, many of the satellite repeats are accumulated in the “junk box” that appears in the so-called "unplaced chromosome" of the assemblies of most genome sequencing projects. This is the situation of bread wheat, whose genome has been sequenced, assembled, and characterized (IWGSC Ref-Seq v2.1, https://www.wheatgenome.org) and for which the polyploid nature of its genome complicates the picture for a complete and correct satDNA assembly. Thus, satDNA included for example in telomeric and (peri)centromeric regions was not fully or properly assembled as revealed in our study, confirming the limitation of the sequencing projects in identifying satDNA loci, which appear as “black holes” of the genome (Henikoff and Malik 2002; Kapustová et al. 2019).

In this paper, we have combined the use of RepeatExplorer2/TAREAN (Novák et al. 2010, 2013, 2020) and satMiner pipelines (Ruiz-Ruano et al. 2016) to increase the likelihood of finding new rare satDNA families in wheat in addition to other more abundant satDNAs, which together with a robust cytogenetic analysis have allowed the characterization of the bread wheat satellitome. We have also found the usefulness of the assembled genome in detecting some loci which were not visible by FISH since they were composed by just a single or few repeats of a satDNA family under the detection limit of the in situ hybridization. The deep analysis performed in this work for the identified satDNAs in the wheat assembled genome using BLAST, contributed enormously to shed light on the complex wheat genome organization. Thus, the combined use of all three data sources, complementing each other, has proved enormously satisfactory to have a comprehensive view not only of the satDNA complete set of this species but also of its organization and evolution.

In total, we have identified 34 satDNA families in the *T. aestivum* genome. Of these, to the best of our knowledge, 21 have been identified for the first time in this work. Our results suggest that the satellitome of *T. aestivum* is very complex. We have identified centromeric, subtelomeric and interstitial satellites depending on the chromosome visualization by FISH. Regarding the completeness of the satellitome, it must be said that under our literature review two additional satellites have been described in addition to those studied here (Kapustová et al. 2019). They are two very low abundant satellites according to our analysis (0,0013 and 0,0003% of the genome, respectively) and are found forming a single locus in the subtelomeric region of the chromosome 7D. Thus, we could therefore say that the bread wheat satellitome is composed of at least 36 different satellites.

### Wheat satellitome analysis turns the concept of satellite DNA upside down

Most wheat satellites have an organization that departs from the classical view of satellite DNAs. In addition to classical satDNA loci visible as conspicuous FISH bands, most wheat satellites are scattered through the genome forming short tandems not visible by FISH which responds to a model of generation of different satDNA families from any genome sequence through a birth-dissemination-clustering process (Ruiz-Ruano et al. 2016), in turn, compatible with the library hypothesis (Fry and Salser 1977), as it has been observed in the last few years in other species after the advent of genomics approaches (reviewed in Garrido-Ramos 2021).

In contrast to the classical concept of satellite DNA, we still have a trickier observation. A few wheat satDNAs raise doubts regarding their nature. They have been identified as such using RepeatExplorer2/TAREAN/satMiner and are among the most abundant in the genome. However, the FISH pattern of TaeSat01-584, TaeSat05-500, TaeSat06-403, TaeSat11-506 and TaeSat21-1590 repetitive DNA families is scattered, coinciding with the pattern in the assembled genome sequence in the IWGSC CS RefSeq v2.1 database. Note that in the case of the TaeSat01-584 and TaeSat06-403 families, for example, regions of dense blocks of FISH signal are observed on some chromosomes over the sparse pattern. Although such blocks are compatible with some classical FISH observations of certain satellites, by definition we would be dealing here with highly repeated sequences that cannot strictly be called satellite DNA. A fine analysis of the bread wheat genome assembly revealed that some of these still dispersed sequences are grouped in short tandems in many genome locations and, furthermore, some of them accumulated in certain assembled regions coinciding with regions in which more intense signals appear by FISH (see Table S5 for the case of TaeSat01-584, for example).

It is interesting to note that 18 of the satDNA sequences analyzed in this paper have significant homology with transposable elements (TEs) (Table 4) as has been observed in recent years in multiple satDNAs from different species (Meštrović et al. 2015; Vondrak et al. 2020; Jesionek et al. 2021; Garrido-Ramos 2021). In fact, 12 of the "classic" satDNAs, showing conspicuous FISH bands, derive from TEs, which might support that most likely transposition was involved in their dissemination In fact, the five satDNA families that show a dispersed pattern (Fig. 4 and Table S5), could well represent an intermediate step of the dissemination and amplification processes as they clearly derived from a TE (Table 4). The presence of some short tandems of two or a few more of their repeats at various locations in the genome supports this view. However, with the current genomic results and given that by classical satDNA identification methods (FISH) we would never have identified them, it is strictly difficult to consider them as satDNA properly.

The SF-6 superfamily represents a good example of the process of satDNA dissemination since itis composed of five families that form classic clusters identified by FISH (TaeSat22-320, TaeSat23-319 and TaeSat29-319), a dispersed pattern (TaeSat14-1463) or both (TaeSat25-318). Interestingly, only the longest satDNA of this superfamily (TaeSat14-1463) shows a region (17.3% of its sequence) homologous to a transposon which points to either the repeated sequence of this satellite is a mosaic derived from several sequences (satDNA, CMC-EnSpm transposon and intervening sequences) or that the whole superfamily has diversified from one transposon.

Finally, it should be noted that there are 16 satDNA families that do not show homology with TEs but, like the other 18 satDNAs, have a pattern in which, in addition to conspicuous FISH bands, they have repetitive units scattered throughout the genome, both individually and forming short tandems.

Taking all these observations together we can conclude that the bread wheat satellitome is composed of a large number of satDNA families that have originated from a multitude of different repetitive units that have spread throughout the genome and that constitute the "seeds" for the local amplification that give rise to the generation of classical loci (defined by the ability to be observed as conspicuous FISH bands), an amplification process that appears to be currently active in the bread wheat genome. It is not unreasonable to think that many if not all of these "seeds" come from recombination processes between TEs. This concept of tandemly occurring interspersed repeats as potentially being satDNA "seeds" has recently been suggested by Gržan et al. (2023) to explain observations in the beetle *Tribolium castaneum* and was proposed by Ruiz-Ruano et al (2016) while we find clear evidence here for this model in this paper..

### A diverse and complex catalogue of satDNAs in a huge genome

One of the revelations of this study is the great diversity of satellites existing in the bread wheat genome, in contrast to the previous information we had. This diversity affects the number of different families but also very particularly the organization and abundance of each one as well as the length, bp composition and structure of the repetitive unit of each family. In bread wheat we have not found a relationship between abundance and organization There is no correlation either in the bread wheat satellitome between repeat-unit length and AT-richness as found previously in some satellitomes, in which longer satellites are older and, in turn, they are richer in AT (Ruiz-Ruano et al. 2016, 2019). Notwithstanding, it can be assumed that the longer satellites in bread wheat are older than the shorter ones because the repeat units of all satDNAs are composed of smaller subunits, suggesting an origin of these repeats from shorter sequences, as in other satDNAs (Garrido-Ramos et al. 1995; de la Herrán et al. 2001, 2008; Navajas-Pérez et al. 2005; Macas et al. 2006; Emadzade et al. 2014) which would also explain the common origin of the different members of the different superfamilies from the diversification of pre-existing sequences of shorter size (Navajas-Pérez et al. 2005; Ruiz-Ruano et al. 2019).

There is a positive correlation between intra-family divergence and DP as well as a negative correlation with RPS as expected since DP assists as an index of sequence degeneration being proportional to the time since the last amplification event, while RPS is an index of homogenization (Camacho et al. 2022). Several satDNAs share similar DP values but differ in their divergence values, and vice versa, which suggests different turnover rates for different satDNA families, something that is related to the structure, organization and location of each satDNA family (Navajas-Pérez et al. 2009; Garrido-Ramos 2017, 2021) and probably, in this case, to the parental origin of the satDNA in question. According to our estimations, most, if not all, of the amplification events that gave rise to FISH loci currently observed in the bread wheat genome might have occurred in the diploid or tetraploid parents of *T. aestivum*. All but three satDNAs, appear scattered throughout the genome forming part of chromosomes of the three wheat subgenomes: A subgenome (possibly *T. urartu*), B subgenome (derived from a diploid ancestral of the genus *Aegilops*, possibly *A. speltoides*) and D subgenome (derived from a diploid ancestral related to *T. tauschii*) (Middleton et al. 2014). However, each satellite has been differentially amplified in all three or just in some of the subgenomes as FISH-visible loci and such amplification might have already occurred in the parental genomes. In fact, our results suggest that these thirty satellites seem to exist or to have existed in all the species mentioned either as the germ of future large FISH-visible loci or as already amplified FISH-visible loci or both. On the contrary, families TaeSat27-72 and TaeSat31-889 seem exclusive of the subgenome B, and families TaeSat33-54 and TaeSat34-175 seem exclusive of A and D subgenomes. Therefore, all the bread wheat satDNAs may also be present in the genome of *T. turgidum* (tetraploid parental species of bread wheat composed of A and B subgenomes). It will be very interesting to analyse in the future the distribution by species and the organization within each species of these satDNAs. In this regard, it is striking that some satDNAs, still scattered throughout the three subgenomes, are amplified giving conspicuous FISH bands in only one or two subgenomes, as (TaeSat32-528 in the A subgenome or TaeSat16-567, TaeSat23-319, TaeSat26-210, TaeSat27-72 and TaeSat31-889 in the B subgenome). These loci of these six satDNAs are therefore expected to be present in *T. turgidum* whilethose of TaeSat03-2619 and TaeSat12-369 would be specific to the D genome and should not be in the *T. turgidum* genome.

### Functional significance of the wheat bread satellitome

SatDNAs are a fundamental component of centromeres and pericentromeres as well of the subtelomeric regions, place where they would play several roles (Garrido-Ramos 2015, 2017, 2021). Concerning centromeres, a structural role that supports the functional basis of satDNAs in the centromere of eukaryotes is undoubted (Hartley and O’Neill 2019), a role that could be played by any repetitive sequence with particular sequence-mediated specific stereo-spatial properties and as such with the capacity to acquire spatial configurations such as cruciform structures and other non-B DNA forms (Kasinathan and Henikoff 2018). In this context, any satDNA of a satellitome could, when located in the right place, assume that role being recruited for such function depending of its capacity for the formation of non-B DNA conformations or other properties (Camacho et al. 2022). In fact,, we have not found significant differences between wheat satDNAs located both in centromeric regions or other locations for parameters such as DNA curvature, dyad symmetries or -dG values. Future research on bread wheat parental species could determine whether different satDNA families are replacing each other in the centromeres by differential amplification between species as occurred in grasshoppers (Camacho et al. 2022) or in Fabeae (Ávila Robledillo et al. 2020). There are two (peri)centromeric satDNAs (TaeSat14-1463 and TaeSat28-543) that are candidates to play a centromeric role in all chromosomes of this species. In addition, two other satDNAs (TaeSat16-567 and TaeSat31-889) would be specific to the centromeres of the chromosomes of the B subgenomes which, together with some quantitative differentiation between chromosomes of different subgenomes for some of these satDNAs (Fig. 3), may provide some subgenomic compartmentalization. Interestingly, TaeSat14-1463 is related to transposons (Table 3), supporting the relevant role in centromere function that TEs play in many plant species(reviewed in Garrido-Ramos 2021), as occurs in the wheat *T. boeoticum* (A subgenome) centromeres (Liu et al. 2008) and the centromere of *T. aestivum* chromosome 3B (Li et al. 2013). The mapping of *T. aestivum* centromeres revealed that TaeSat16-567 and TaeSat28-543 (see Table S1) are functionally active on a few chromosomes of the three subgenomes, with TaeSat16-567 being in all B chromosomes (Su et al. 2019). FISH results of this study were somewhat contradictory with our results but comparable. Curiously, Su et al. (2019) found that, in addition to these two satellites, four Ty3/Gypsy elements were part of the functional centromere of all chromosomes of all subgenomes of the specie. In fact, extensive retrotransposon amplification might have occurred in the pericentromeric regions during the speciation of the diploid and polyploids wheat species which might undergone dynamic changes inserting differentiated centromeric retrotransposons in such a way that hexaploid wheat subgenomes might have their own specific (peri)centromeric elements, supporting that centromeres and pericentromeres are critical regions for subgenome differentiation in polyploids (Liu et al. 2008; Li et al. 2013). Interestingly, phylogenetic analysis revealed also that the TaeSat16-567 and TaeSat28-543 satellites have diverged in the three *T. aestivum* subgenomes (Su et al. 2019).

Differentiation at the subtelomeric level might also be one of the key aspects in the possible functions of satDNA sequences (Calderón et al. 2014; Aguilar and Prieto 2020). The evolution of repeated DNA sequences located on the subtelomeres of the wheat B genome have been previously studied (Salina et al. 2009). Our study has revealed that the subtelomeric regions of hexaploid wheat are highly polymorphic for the satDNAs identified, supporting the dynamic nature in the subtelomeric regions and their putative implications in specific homologous recognition and pairing. The fact that many of the satDNAs were specifically found at the subtelomeric chromosome regions might suggest a functional role on chromosome specificity during early meiosis in a polyploidy such as wheat. In fact, we have found 10 satDNAs exclusive of wheat subtelomeres. These satDNAs might joint specific proteins or provide a designed DNA conformation that might contribute to correctly distinguish homologous chromosomes from their related or homoeologous chromosomes and allow specific DNA-DNA homologous interactions or thought the proteins joint to these DNA sequences. In addition, each chromosome has its own profile for the subtelomeric region (Fig. 5), which suggest than these chromosome-specific profiles are good candidates to constitute a solid differentiation basis to restrict chromosome specificity to homologous chromosomes for correct recognition and pairing during meiosis. In fact, previous cytological observation indicated that initiation of homologous chromosome synapsis and further recombination occurred at the chromosome ends (Prieto et al. 2004b), finding terminal chiasmata at the subtelomeric regions in all wheat bivalents and failing recombination when subtelomeres are absent (Calderón et al. 2014; Scherthan 2001; Schwarzacher 2003). At the beginning of meiosis, when telomeres are attached to the nuclear envelope and shaking to find the right partner to associate with, homologous chromosome regions of high affinity must compete for zippering with homoeologous or equivalent regions from the homoeologous chromosomes with lower affinity. When the affinity among the distal regions of homologous chromosomes is strong enough to oppose shaking, zippering of only those true homologous chromosomes is allowed. Subtelomeres play a key role in this homologous chromosome search and satDNA sequences included in subtelomeres might facilitate homologous chromosome identification and pairing. The high variability found in this work among homoeologous chromosomes for the satDNA sequences included in the subtelomeric regions might increase specificity for chromosome recognition and pairing in a polyploidy such as wheat. This fact, together with the results found for the (peri)centromeric regions, pave the way for further studies on the implication of satDNAs in chromosome recognition and pairing during meiosis in wheat. This work also contributes with the identification of several new satDNAs as potential targets to be studied for shedding more light into the knowledge of the subtelomeric DNA sequence and three-dimensional structure, both critical for the initial chromosome interactions at the onset of meiosis in wheat.

## Experimental procedures

### Plant material and growing conditions

Hexaploid (bread) wheat *Triticum aestivum* L., cv. Chinese Spring (CS) (2n = 6x = 42) was used in this work to perform genomic and cytogenetic analyses. Seeds were germinated in the dark at 25 °C on wet filter paper in Petri dishes for 2 days and then transferred to pots and grown in the greenhouse at 24 ± 2 °C under long days photoperiod.

### Sequence analysis

Genomic DNA (gDNA) was isolated from wheat leaves using the standard CTAB procedure relative to (Murray and Thompson 1980) with some modifications according to (Hernández et al. 2001). A NanoDrop1000 spectrophotometer (NanoDrop Technologies, USA) was used to determined DNA concentration and quality.

Next Generation Sequencing was carried out at Macrogen Inc. (Macrogen Inc., Seoul, Korea) based on Illumina NovaSeq 6000 150PE (2 × 151 bp), yielding about 21 Gb (~ 1.2 × coverage) data. Illumina sequencing data can be accessed at SRA-Genbank database in the BioProject PRJNA856123.

### SatDNA mining

We applied the protocol satMiner (Ruiz-Ruano et al. 2016), which is based in consecutive rounds of clustering of Illumina reads by RepeatExplorer 2 (Novák et al. 2013, 2020), using a subset of reads (2,000,000 per library), and subsequent filtering of the already assembled reads using DeconSeq (Schmieder and Edwards 2011). RepeatExplorer 2 (Novák et al. 2013, 2020) executes an integrated version of the TAREAN tool (Novák et al. 2017), which performs automated identification of satellite DNA repeats based on the topology of their cluster graphs.

We first performed a quality trimming with Trimomatic (Bolger et al. 2014), and randomly selected 2 × 2,000,000 Illumina reads with SeqTK (https://github.com/lh3/seqtk), to run RepeatExplorer2 with default options. Cluster graphs with circular shape were selected using TAREAN which generates a consensus monomer sequence for each satDNA cluster.

We filtered out the reads showing homology with the already clustered contigs and the already identified satDNA using DeconSeq, and selected a new set of 2 × 2,000,000 reads from the filtered libraries, that were clustered with RepeatExplorer2 in a second round. This allows detecting satDNAs being poorly represented in the raw reads. We repeated the filtering using the clusters in the second round, and selected 2 × 2,000,000 reads for three additional rounds. Performing additional rounds of clustering and filtering have shown to be highly successful as it allows detecting satDNAs which, due to their low abundance, had gone unnoticed because their signals were masked by those of highly abundant elements (Ruiz-Ruano et al. 2016). After multiple iterations, we performed a similarity search among the sequences with RepeatMasker (Smit et al. 2015) using a custom python script (https://github.com/fjruizruano/ngs-protocols/blob/master/rm_homology.py).

### Repeat landscapes

In order to estimate abundance and divergence for each identified satDNA, we aligned 2 × 10 millions of randomly selected read pairs to the consensus sequences in the resulting satDNA database, using RepeatMasker with a publicly available script (https://github.com/fjruizruano/satminer/blob/master/repeat_masker_run_big.py). We used the calcDivergenceFromAlign.pl built-in tool of RepeatMasker to obtain a histogram of the Kimura 2-Parameter divergence for each element. Next, we transformed the abundance values to express them as genome proportion by dividing the number of aligned nucleotides by the total number of nucleotides in the selection of 20 million reads. The resulting histograms (hereafter referred to as Repeat Landscapes, RLs) were plotted. Following (Camacho et al. 2022), we searched for divergence peaks, i.e., those divergence values showing the highest abundance in the repeat landscape (DIVPEAK). Then, we summed up the abundances of all satDNA sequences at ± 2% divergence from the DIVPEAK class to calculate abundance in the 5% peak or PEAK-SIZE to get a collection of sequences diverging 5% or less to the consensus sequence. Finally, we calculated relative peak size (RPS) as the quotient between PEAK-SIZE and total abundance, which measures the proportion of repeat units being part of the last amplification event. RPS serves as an index of homogenization because it is expected to increase with satDNA amplification, as the new units derived from tandem duplication will initially show identical sequences, thus increasing global identity. DIVPEAK serves as an index of degeneration because it will increase by mutation accumulation and is thus proportional to the time passed since the last amplification (Camacho et al. 2022).

### SatDNA sequence bioinformatics analysis

A Basic Local Alignment Search Tool (BLAST®) search was performed for each satDNA in order to establish homologies with other satellites or sequences in the GenBank databases. Likewise, a fine search of these sequences in the wheat genome was carried out using BLAST trailing the International Wheat Genome Sequencing Consortium (IWGSC) CS RefSeq v2.1 assembly of the *T. aestivum* genome through NCBI's Genome Data Viewer (GDW) (Rangwala et al. 2021) in order to identify the locations of each satDNA family within the bread wheat genome (https://www.ncbi.nlm.nih.gov/assembly/GCF_018294505.1). In addition, we searched for homologies with transposable elements with RepeatMasker (Smit et al. 2015) with “no_low” and “no_is” options. The EMBOSS suite of bioinformatics tools (Rice et al. 2000) was used for basic analyses such as the Adenine + Timine content (AT%) or the presence of internal repeats (direct or inverted) as well as palindromes in the consensus satDNA sequences. The programs used from the package were INFOSEQ, MATCHER, ETANDEM, and PALINDROME. Additionally, we checked all satDNAs families to test on the propensity to form non-B DNA conformations using the Mfold web server (http://www.unafold.org/mfold/applications/dna-folding-form.php) for nucleic acid folding prediction (Zuker 2003), estimating Gibbs free energy (dG) of the predicted secondary structures (SantaLucia 1998). We also checked the consensus sequences of satDNAs families for sequence dependent bendability/curvature propensity of repeats. The bendability/curvature propensity plots were made with the bend.it server, http://pongor.itk.ppke.hu/dna/bend_it.html#/bendit_intro, using the DNase I based bendability parameters of (Brukner et al. 1995) and the consensus bendability scale (Gabrielian and Pongor 1996).

### Probes design for cytogenetic validation of satDNA sequences by in situ hybridization

The different satDNAs families were amplified by PCR (“Polymerase Chain Reaction”) using specific primers designed with Primer-BLAST software tool from NCBI (https://www.ncbi.nlm.nih.gov/tools/primer-blast/index.cgi?LINK_LOC=BlastHome) (Table S7). For monomers shorter than 80 bp, primers were designed manually. OligoAnalyzer™ tool (https://eu.idtdna.com/calc/analyzer) was used to confirm the absence of putative secondary structures in the primers sequences (harpins, self-dimers and hetero-dimers). For families of sequences consisting in monomers longer than 80 bp, we performed PCR amplification with the following conditions: a starting denaturation step at 94 °C for 5 min (minutes), 35 cycles at 94 °C for 30 s (seconds), followed by an annealing step of 42–60 °C (primer-dependent, see TableS1) for 30 s, and an extension at 72 °C during 1 min. A final extension step at 72ºC for 6 min was added. In the cases of amplifying satDNAs shorter than 80 bp, we reduced the time of annealing to 10 s in order to get longer amplicons according to (Ruiz-Ruano et al. 2016). PCR products were loaded in 1% agarose gel electrophoresis in 1 × TAE (40 mM TrisBase, 20 mM Acetate and 1 mM EDTA, dH_2_O until the volume of 1 L) running buffer and visualized using Quantity One 1-D Analysis Software Bio-Rad. A 100 bp DNA Ladder ready to Load (Solis BioDyne) was used as a reference for molecular weight DNA. PCR products from short monomers were displayed as a smear in agarose gels and were re-amplified using 1 μL of the previous PCR product in a new PCR mix. For PCR amplification, 20 ng of wheat genomic DNA was used in a total reaction mixture volume of 40 μl using MyTaq™ polymerase(Bioline). DNA amplification samples were sequenced to confirm the reliability of the PCR products.

The satDNAs families sequences were indistinctly labelled by nick translation kit with biotin-11-dUTP (Boehringer Mannheim Biochemicals, Germany) or digoxigenin-11-dUTP (Roche Applied Science, Indianapolis, IN, USA),respectively, according to the manufacturer’s instructions as described previously (Prieto et al. 2004a). Nick translation was performed in a thermocycler (ThermoBrite® Leica) at 15 °C for 90 min.

### Chromosome preparations of root tip cells in somatic metaphase

The protocols used for sample obtaining and treatment were previously described in (Prieto et al. 2001, 2004a). Seeds were germinated on wet filter paper in dark for 5 days at 4 °C, followed by a period of 24 h at 25 °C. Emerging seedling roots with 1–2 cm (centimetres) long were cut, incubated during 4 h in 0.05% w/v colchicine at 25 °C, fixed in 100% ethanol- acetic acid, 3:1 (v:v) and stored at 4 °C until their use. Plants were then grown in a greenhouse under semi-controlled conditions of temperature (25ºC day/15ºC night) and relative humidity (40%). During the summer months, plants were grown in a climatic chamber with controlled conditions of photoperiod (12 h of light), temperature (20ºC day/15ºC night) and relative humidity (60%).

Preparation of chromosome spreads was done as described in (Prieto et al. 2004a) with some modifications. That is, before squashing, roots were washed in 1 × enzyme buffer (4 mM citric acid and 6 mM sodium citrate) 3 times for 5 min each. Them, meristems were cut and incubated during one hour in the enzyme mixture: 0.5% pectolyase Y23 (Kyowa Chemical Products Co., LTD), 1% cellulose “Onozuka” RS (Yakult Pharmaceutical Ind. Co., LTD) and 20% peptinase (Sigma) in dH_2_O.

Due to the difficulty of getting good quality chromosome spreads, in some cases, we have hybridized the same sample with two different satDNAs simultaneously, each one labelled in red or green as described previously.

### Fluorescence in situ hybridization (FISH)

For in situ hybridization experiments, both biotin and digoxigenin labelled probes were mixed to a final concentration of 5 ng/μl in the hybridization mixture (50% formamide, 2 × SCC, 5 ng of each digoxigenin and biotin-labelled probes, 10% dextran sulphate, 0.14 μg of yeast tRNA, 0.1 μg of sonicated salmon sperm DNA, and 5 ng of glycogen). The in situ hybridization protocol was performed according to (Cabrera et al. 2002).

Post-hybridization washes were conducted twice at 2 × SSC (5 min each) at 37 °C followed by another wash in 1 × SSC at room temperature (RT). Biotin- and digoxigenin-labelled probes were detected with streptavidin-Cy3 conjugates (Sigma, St. Louis, MO, USA) and antidigoxigenin FITC antibodies (Roche Diagnostics, Meylan, France), respectively. Wheat chromosomes with positive signals for the satDNAs sequences were identified based on patterns of the repeat sequences pAs1 (Rayburn and Gill 1986; Cabrera et al. 1995) and GAA (Pedersen et al. 1996; Pedersen and Langridge 1997). Total DNA was counterstained with 4′,6-diamidino-2-phenylindole (DAPI) and mounted in Vectashield (Vector Laboratories, Burlingame, CA, USA). Hybridization results were visualized using a Nikon Eclipse 80i epifluorescence microscope and images were captured with a Nikon CCD camera using the Nikon 3.0 software (Nikon Instruments Europe BV, Amstelveen, The Netherlands). Finally, images were processed with Photoshop 11.0.2 software for adjustment of brightness and contrast (Adobe Systems Inc., San Jose, CA, USA).

### Supplementary Information

Below is the link to the electronic supplementary material.

Supplementary file1 (PDF 21048 KB)

Supplementary file1 (XLSX 40 KB)

Supplementary file1 (XLSX 23000 KB)

## Data Availability

All raw data underlying this article are available in the article.
