# Supplementary material for: Bread wheat satellitome: a complex scenario in a huge genome
Source: Plant Mol Biol. 2024 Jan 30;114(1):8. doi: 10.1007/s11103-023-01404-x (PMC10827815; doi:10.1007/s11103-023-01404-x)

Supplementary Information

Bread wheat satellitome: A complex scenario in a huge genome

Ana Gálvez-Galván, Manuel A. Garrido-Ramos, Pilar Prieto

Contents

Supplementary Tables

Supplementary Table S1. SatDNA families previously published in other studies……………...1

Supplementary Table S3. Internal inverted subrepeats for each satDNA family…………….....2

Supplementary Table S4. Prediction of curvature for each satDNA family……………………3

Supplementary Table S6. Presence of satDNA repeats in subtelomeric regions (500 Kb from each end of every chromosome) using the assemblies IWGSC (CS) John Hopkins (CS) and HIFI-PACBIO (Fielder). The different subgenomes are differentiated by colour (A=blue; B=pink; D=green). The presence of each satDNA was checked on the 500Kb termini from each chromosome end (S: short arm; L: Long arm)……………………………………………………4

Supplementary Table S7. Primer designed in this study to amplify each satDNA family………7

Supplementary Figures

Supplementary Figure S1. Sequence in fasta format for each satDNA family……………….…….8

Supplementary Figure S2. Repeat landscapes plots for satDNA families in bread wheat (*T. aestivum* L.). For each satDNA the abundance (Y axis) and the divergence (X axis) with respect to the consensus sequence was built according to the satDNA repeat unit………………………14

Supplementary Figure S3. Secondary structure prediction for satDNA Taesat21-1590 dG= -301.78; TaeSat14-1463 dG= -170.87; TaeSat01-584 dG= -97.28; TaeSat16-567 dG=-60.15; TaeSat28-543 dG=-55.27; TaeSat12-369 dG=-34.59…………………………………………...15

Supplementary Figure S4. Alignment of the sequences belonging to superfamily 1 (SF-1): TaeSat04-337, TaeSat07-343 and TaeSat10-206. The duplicated AAATGATGAT sequence is tagged in yellow………………………………………………………………………………16

Supplementary Figure S5. Alignment of the sequences belonging to superfamily 2 (SF-2): TaeSat05-500 and TaeSat11-506……………………………………………………………..17

Supplementary Figure S6. Alignment of the sequences belonging to superfamily 3 (SF-3): TaeSat08-663 and TaeSat19-653…………………………………………………………….…….18

Supplementary Figure S7. Alignment of the sequences belonging to superfamily 4 (SF-4): TaeSat09-335 and TaeSat20-322…………………………………………………..…………19

Supplementary Figure S8. Alignment of the sequences belonging to superfamily 6 (SF-6): TaeSat22-320, TaeSat23-319, TaeSat25-318 and TaeSat29-319………………………………..20

Supplementary Figure S9. Alignment by pairs of the sequences belonging to superfamily 6 (SF-6): TaeSat22-320, TaeSat23-319, TaeSat25-318 and TaeSat29-319…………………………………………………………………………………….…...21

Supplementary Figure S10. Idiograms of chromosomes of *T. aestivum* cv. Chinese Spring bread wheat representing satellites DNA location identified in this work by fluorescent *in situ* hybridization (FISH). (a) satDNAs with terminal location (telomeric and subtelomeric); (b) satDNAs with multiple locations (terminal, centromeric and interstitial), and (c) satDNAs with (peri)centromeric location……………………………………………………………………….23

Supplementary Tables

Supplementary Table S1. SatDNA families previously published in other studies.

SatDNA	homologous satDNA		FISH	
	Complete sequence	Partial sequence	FISH in *T. aestivum* cv. CS[a]	FISH in other species
**TaeSat02-118**		(pTa-86, pTa-83, pTa-505)[1], TaesatDNA[2], pSc119.2[11]	Validated	
**TaeSat03-2619**	TaeCsTr99[3]		Validated	
**TaeSat04-337**	pAs1[10]	(pTa-173, pTa-535, pTa-s53)[1], pTa1[4], Afa family[5]	Validated	*Aegilops squarrosa*
**TaeSat05-500**		pTa-451[1]		
**TaeSat07-343**	pTa-535[1]	pAs1 related [10], (pTa-173, pTa-s53)[1], Afa family[5]	Validated	
**TaeSat09-335**	pTa-465[1]		Validated	
**TaeSat10-206**	pTa-173[1]	pAs1 related[10], (pTa-535, pTa-s53)[1], pTa1[4], Afa family[5]	Validated	
**TaeSat11-506**		pTa-451[1], TaeCsTr111[3]		
**TaeSat13-44**	Ta-3A1[6]		Validated	*T. monococcum*; *Ae. Speltoides; Ae. Longissima; Ae. Searsii*; *Secale cereal*; *Ae. Tauschii*; *T. durum*; *T. carthlicum*; *T. dicoccoides; Thinopyrum ponticum; Dasypyrum villosum*; *Th. intermedium*
**TaeSat16-567**	(pTa-1, pTa-k18)[1], CentT566[7]	TaiI family[8]	Validated	*Ae. Speltoides; Ae. tauschii*
**TaeSat22-320**		(p451, p441)[9]		
**TaeSat25-318**		(p451, p441)[9]		
**TaeSat28-543**	CentT550[7]			*Ae. Speltoides; Ae. tauschii*

Supplementary Table S3. Internal inverted subrepeats for each satDNA family.

Satellite Name	Length	Max. length Inverted repeats	Number of inverted repeats < 10 pb	Number of inverted repeats > 10 pb	dG
**TaeSat01-584**	584	16	161	51	-97.28
**TaeSat02-118**	118	7	3	0	-12.04
**TaeSat03-2619**	2619	13	3378	940	-185.87
**TaeSat04-337**	337	25	89	20	-30.5
**TaeSat05-500**	500	16	103	24	-46.77
**TaeSat06-403**	403	16	71	19	-32.97
**TaeSat07-343**	343	20	53	18	-33.14
**TaeSat08-663**	663	21	284	88	-68.66
**TaeSat09-335**	335	15	49	10	-58.05
**TaeSat10-206**	206	20	20	6	-13.72
**TaeSat11-506**	506	15	146	46	-48.45
**TaeSat12-369**	369	13	63	19	-34.59
**TaeSat13-44**	44	10	3	1	-6.24
**TaeSat14-1463**	1463	22	968	333	-170.87
**TaeSat15-620**	620	20	189	66	-54.27
**TaeSat16-567**	567	15	140	44	-60.15
**TaeSat17-323**	323	38	69	26	-47.69
**TaeSat18-733**	733	15	268	72	-57.47
**TaeSat19-653**	653	17	306	67	-79.43
**TaeSat20-322**	322	12	39	10	-49.25
**TaeSat21-1590**	1590	23	1232	423	-301.78
**TaeSat22-320**	320	11	75	14	-44.23
**TaeSat23-319**	319	15	41	15	-54.61
**TaeSat24-338**	338	18	54	20	-61.06
**TaeSat25-318**	318	12	51	10	-44.21
**TaeSat26-210**	210	20	17	9	-13.8
**TaeSat27-72**	72	6	1	0	-5.6
**TaeSat28-543**	543	13	101	26	-55.27
**TaeSat29-319**	319	20	45	15	-46.44
**TaeSat30-1389**	1389	22	537	162	-126.12
**TaeSat31-889**	889	16	302	77	-88.13
**TaeSat32-528**	528	15	95	24	-57.36
**TaeSat33-54**	54	10	2	1	-0.38
**TaeSat34-175**	175	15	10	4	-5.02

Supplementary Table S4. Prediction of curvature for each satDNA family.

Satellite Name	Length	Abundance (%)	Divergence	AT content (%)	SF	Max. Peak Curvature (Position)
**TaeSat01-584**	584	0.4729	0.0982	37.33		6.5 (370)
**TaeSat02-118**	118	0.3053	0.1253	48.31		12 (85)
**TaeSat03-2619**	2619	0.2757	0.2257	63.38		12.5 (750)
**TaeSat04-337**	337	0.271	0.0813	65.28	SF-1	12 (205)
**TaeSat05-500**	500	0.2204	0.2402	56.8	SF-2	12 (175)
**TaeSat06-403**	403	0.1815	0.2185	60.55		10 (60)
**TaeSat07-343**	343	0.1688	0.0667	59.18	SF-1	10.5 (70)
**TaeSat08-663**	663	0.1066	0.0711	65.16	SF-3	16 (590)
**TaeSat09-335**	335	0.1003	0.0972	36.12	SF-4	7 (170)
**TaeSat10-206**	206	0.0892	0.0579	65.62	SF-1	10.5 (100)
**TaeSat11-506**	506	0.0784	0.1244	62.85	SF-2	12 (390)
**TaeSat12-369**	369	0.046	0.0508	60.16	SF-5	8 (220)
**TaeSat13-44**	44	0.0348	0.0761	70.45		
**TaeSat14-1463**	1463	0.0261	0.1825	59.13	SF-6	12 (1020)
**TaeSat15-620**	620	0.026	0.2466	62.58		12 (130)
**TaeSat16-567**	567	0.0199	0.1161	53.62	SF-5	12.2 (390)
**TaeSat17-323**	323	0.0143	0.089	62.23		11 (140)
**TaeSat18-733**	733	0.0121	0.0792	66.58		16 (550)
**TaeSat19-653**	653	0.0111	0.177	54.52	SF-3	13 (380)
**TaeSat20-322**	322	0.0096	0.1574	39.75	SF-4	6.5 (90)
**TaeSat21-1590**	1590	0.0091	0.0659	35.6		10 (980)
**TaeSat22-320**	320	0.0079	0.0879	48.12	SF-6	11.8 (275)
**TaeSat23-319**	319	0.0075	0.1082	42.01	SF-6	15 (75)
**TaeSat24-338**	338	0.0069	0.1338	40.83		8 (170)
**TaeSat25-318**	318	0.0048	0.1595	45.6	SF-6	13 (190)
**TaeSat26-210**	210	0.0045	0.0324	59.52		8 (130)
**TaeSat27-72**	72	0.0044	0.0829	50,00		1.05 (35)
**TaeSat28-543**	543	0.004	0.1078	55.43		15.8 (140)
**TaeSat29-319**	319	0.0035	0.1039	47.02	SF-6	11.8 (55)
**TaeSat30-1389**	1389	0.0032	0.1373	63.43		14 (90)
**TaeSat31-889**	889	0.0029	0.028	59.39		10 (220)
**TaeSat32-528**	528	0.0019	0.0237	56.63		10 (240)
**TaeSat33-54**	54	0.0006	0.2204	72.22		
**TaeSat34-175**	175	0.0004	0.1621	70.69		9 (50)

Supplementary Table S6. Presence of satDNA repeats in subtelomeric regions (500 Kb from each end of every chromosome) using the assemblies IWGSC (CS) John Hopkins (CS) and HIFI-PACBIO (Fielder). The different subgenomes are differentiated by colour (A=blue; B=pink; D=green). The presence of each satDNA was checked on the 500Kb termini from each chromosome end (S: short arm; L: Long arm).

	**IWGSC (CS)**																				
**satDNA family**	1A	1B	1D	2A	2B	2D	3A	3B	3D	4A	4B	4D	5A	5B	5D	6A	6B	6D	7A	7B	7D
TaeSat01-584	S	S	SL	SL	S	S		S	L			L	SL	SL	SL			SL	SL	S	S
TaeSat02-118		L	L		S	SL			L	L	SL	S		S	S		L	L	S		L
TaeSat03-2619														L							
TaeSat04-337		SL	SL	L	SL	SL	L	SL	SL	L	L	SL	L		S	SL	SL	SL	SL	L	SL
TaeSat05-500																					
TaeSat06-403	SL			S		L				S	S		SL		S	L		L		S	
TaeSat07-343			S		S		L	L			L						SL		L	S	
TaeSat08-663			S				S		S	SL			L	S		SL	SL		S		
TaeSat09-335							S		L									S			
TaeSat10-206		S	SL	L	SL	SL	L	SL	SL	L	L	SL			S	SL	SL	SL	SL	L	SL
TaeSat11-506															L						
TaeSat14-1463										L											
TaeSat15-620							S			SL			L			SL					
TaeSat16-567																S	S				
TaeSat17-323			S				S			L											
TaeSat18-733							S		S			S									
TaeSat20-322						L															
TaeSat21-1590																					
TaeSat23-319																					
TaeSat25-318								S						S							
TaeSat24-338				L																	
TaeSat26-210				S						SL	S		L								
**Telomeric DNA**	S	L						L		S						SL	S		S	S	SL

	JOHNHOPKINS (CS)																				
**satDNA family**	1A	1B	1D	2A	2B	2D	3A	3B	3D	4A	4B	4D	5A	5B	5D	6A	6B	6D	7A	7B	7D
TaeSat01-584		S	L	S		SL				S			S	SL	SL	L	S	SL	S	SL	S
TaeSat02-118		L	L		S			S		L	SL	S		SL				L	S		
TaeSat03-2619																S					
TaeSat04-337		SL	SL			SL	SL	SL	SL	L	L	SL	L	S	L	SL	S	SL	SL	S	SL
TaeSat05-500									L												
TaeSat06-403	L			SL						S	S		S			L		L		S	L
TaeSat07-343					S		L	S	S		L	L	S		L	L	S			SL	L
TaeSat08-663			S	L			L	L		SL			L	S	S	S			S	L	
TaeSat09-335					L	S	S						S	S				S		L	L
TaeSat10-206		S	SL			SL		SL	SL	L	L	SL		S	L	L	S	SL	SL	S	SL
TaeSat11-506																					
TaeSat14-1463										L										L	
TaeSat15-620										SL			L			S					
TaeSat16-567																S					
TaeSat17-323										S			S				S			S	
TaeSat18-733												S									
TaeSat20-322					S								S								L
TaeSat21-1590																					
TaeSat23-319						L															
TaeSat25-318																					
TaeSat24-338					L																L
TaeSat26-210										SL											
**Telomeric DNA**	SL												S					L	L		

	HIFI-PACBIO (Fielder)																				
**satDNA family**	1A	1B	1D	2A	2B	2D	3A	3B	3D	4A	4B	4D	5A	5B	5D	6A	6B	6D	7A	7B	7D
TaeSat01-584	S	S	SL	S	L	SL	SL	L		S	SL	SL	L	L	L		S			S	
TaeSat02-118		L	S		L	S		S			SL		S	S			L				
TaeSat03-2619																					
TaeSat04-337		S	SL	L		SL	SL	L		L		L	L		SL	SL	L	SL		S	SL
TaeSat05-500																					
TaeSat06-403	L			SL		L							L			L			L		
TaeSat07-343		S	L	L			S	S	S							SL			S		S
TaeSat08-663		L	S	L				SL					L			S	L			S	
TaeSat09-335			L						SL								S				
TaeSat10-206		S	SL			S	L	L	S			L			SL	S	L	SL			SL
TaeSat11-506		L													L						
TaeSat14-1463						L						L					S				S
TaeSat15-620			S	L									L			S					
TaeSat16-567																					
TaeSat17-323				L		L	S					L				L	S				S
TaeSat18-733																					
TaeSat20-322																					
TaeSat21-1590								L													
TaeSat23-319																					
TaeSat25-318																					
TaeSat24-338					L																
TaeSat26-210													L							L	
**Telomeric DNA**	SL			S			L		L				L		L						L

Supplementary Table S7. Primer designed in this study to amplify each satDNA family.

satDNA	Forward	Reverse	T annealing (°C)
**TaeSat01-584**	CAGAATAACAGGGGGTGTGG	GCCGTAGTGCTTCTTCAACC	60
**TaeSat02-118**	GTTTTGGGGTCCCGGAGT	CACGAAACGGGCCAGAAT	55
**TaeSat03-2619**	AGCCTTCGAACGACCCTTTT	GGAGACGTGTCACCCTGTTC	55
**TaeSat04-337**	ACCATGCCAACTTTCAACCT	ATGCACCATTCAAAGCCACA	55
**TaeSat05-500**	AATCACTGCCTCTTTTCGCC	AAACATGCACCCAAGGACAC	55
**TaeSat06-403**	TGTTTTGCGGATGGTCAGTA	CATGGCAAGAACAAGTGCAT	60
**TaeSat07-343**	CGTGTTGGAAATTGATGACG	TGTTGGAACTTGGCATGGTA	55
**TaeSat08-663**	CCAACTGAATCGGCGGAAAT	CAACCACTAGCTAGGAACGGT	60
**TaeSat09-335**	ATCTTGCTGTGGGTCTGGAG	CGCTCCGGTGGCATTCTA	55
**TaeSat10-206**	GTTGAGAGGGTTACGGCAAA	GGCTTTGAATGGTGCATTTT	60
**TaeSat11-506**	TCATGCCACCAAGATGATGTG	CATGTGCCCATGCCTTGAGA	55
**TaeSat12-369**	TGAGGTCTCTTGCATTTCCCT	ACTACTTGTGGAGCCAAAACG	60
**TaeSat13-44**	GAACTAGCTCTATAAGCTAGT	AACTCTAGTGTAAAATTATTT	42
**TaeSat14-1463**	AGGGGACGAGAGTTAGGCAA	GGATCATTTGGTGGGACCGT	55
**TaeSat15-620**	CCTTCACAATGCTTCTAGGTGG	CCTCCAAATTGCAGCTCAGTC	55
**TaeSat16-567**	TTGCCCCAAAATTTCTTGTC	TCTATGATCCGGGCAATCTC	55
**TaeSat17-323**	AGGGGCCATTTGGTCTAACT	TATACCTCAAGGGGGCATTG	55
**TaeSat18-733**	GGACTACCCTCGAAGCATGA	TTCCACCAGCATTTCTTCCT	55
**TaeSat19-653**	ATGCGTTTTTCGTGAAGCGG	CATCACAAGACGGAGGCATG	60
**TaeSat20-322**	GTCTACGCATGCCAGAACAC	TCTAGTGTTGGGTTGGGTCC	55
**TaeSat21-1590**	GCTTCCGTCTTGGATCGCTA	GGGCAAGAGGGAAGGGATTT	55
**TaeSat22-320**	TATGAAGGATGTCAGCCCCG	AGTGTTTTCGGTGCGTTTCA	60
**TaeSat23-319**	CGACGCTGTACGGATTTTCT	CAAGTGCCAGTTCGGTTTG	55
**TaeSat24-338**	GTGCTCGATATAGAGACCGCC	GGTCTAGGAAGTTGACCGGC	50º/ 55º/ 60º
**TaeSat25-318**	GGCAGACCGACAAGTAGCTG	TTCTCCGTCAAACGTCACGA	55
**TaeSat26-210**	GGAGGTCCTGTGTTCAATTCC	GGATTGCGTCCTTTTCTTGGTT	55
**TaeSat27-72**	CAATCTCAAGATGTTGTGTCGG	GAAGTCACCACATACGCCTT	52
**TaeSat28-543**	TTGTGATTGTTTTGGCGGGT	CACCCTCACGTTTTCACCAG	55
**TaeSat29-319**	GTCGAAAATAGGCGTGCGAG	GTCGAAAATAGGCGTGCGAG	60
**TaeSat30-1389**	CATCATGTGCGGTCGGATG	TTGTACTGTGTTGCGTTGTGC	60
**TaeSat31-889**	GACAGCCAGCAGAAACAACA	AGGCAGATAGTTGTTGGGGG	60
**TaeSat32-528**	GAGCGTGCACTAAAAGAGGG	CCGTTCTACTTCCTCCCTCC	60
**TaeSat33-54**	TAAAATACAGTATTGCGATTAT	GTACTGAAATTAGTGATATAA	42
**TaeSat34-175**	CAAGTTTTTAATTCACGCAAATC	CCAGGTGAACATATTTTAATGAACTG	60

Supplementary Figures

Supplementary Figure S1. Sequence in fasta format for each satDNA family.

>TaeSat01-584
AATAAGGAGGCACTTCCTTGCGGCGGCCGTGGACCCAGCTGTCAGCCTCTCCACGTACAGTACACTTCC
GATGGAAGTCGTTCCTTGACCACGTTGACCACGCCGCGCCGAGAGCACCAGGGCGGTGGACGACGGCG
AGGCCTAGGAAGGGGACGACGCGGAGCCGGGGAAGACGCGGCAGTGGATGCCCACGCGGAGAGGAGT
ACGAGGGTTCACTGGTTCGGCTGCGGTGTGAGGCTGCCGTCGCCGCAGAATAACAGGGGGTGTGGGTG
AGTAGAGGGATGGCCTGGCCAGCGGTGGGAGTAGTAGGGGGCGGTGAGGCCTCCGCGGCAGCACAGC
CGGCCACGGGAGGCAGGAGCAGGCGGCACGACCGGCGCTGCTTTGGGCGGCTGGAGCAAGAAGACCA
GAGGTTGAAGAAGCACTACGGCCGTTGGATGGACATCGTACGGTCACTGGAGCTAGAATCGTTCATATT
GACTAAGTTGACAAAGCCCTCCGTCCCCGTCAACTTAGTAGGCCCACAAGTCAGCCTCCCACCATGGTG
GGTCCCAGCTAGCAGGGGGAGTATTCATTTTTTTGTGCGT

>TaeSat02-118
CTGTTTTGGGGTCCCGGAGTGATTTCCATGATTGACGAACCCCAAGGTGCGCTTACGTGTCGGTCATCA
ACACTCACAGTTTTGGCCGATTCTGGCCCGTTTCGTGGACTATTACTCA

>TaeSat03-2619
GGTTGATCATTAGCAATTGAGGGTCCTTACAATCGGTATTACTATGAGGATTACATGGATTGTCGTGAA
ACAGATACACTAGAACCATAAGTGTATGAAAGCTCTCCTAATGTTACTAACTTGGTGCGGATCAAACTT
GCTTACTCATGACGACATCAAGCATTTGAGAAAGCTCGTCGACGGAATATACGAGCCAATTTATGTAAT
GTAAAAAATCCTACTAGCATATGAAATGAATAATATGAAAACTCTATATTTGAAATACATGGTGCTACT
TTGAAGTACAAGTGTGTATAAAGGATAGTATAGTGTGCCTCCTCTCTCCTTTTCTCTCTTTTTTATTTTTT
TTTCTCTTTTTCTGGTGGGCTCTTTGACCTTTCTTGGTGCGGTAGATGTGGCGAAGTAGTAGATCCAACA
AACGAGGAACCACCGAAAGCTGACGCCGGCATGACCGAAACTGGCAAAGAAACCATGTAGCAGCATA
TGGCGATATCTTCACAACTGCTAGTTGTAGAGGCCAAGTTCATGAAATTTCAGTGGCAGCCTTCGAACG
ACCCTTTTCCACTAATGACAGAAACCAAATTTTTCGCAATTAATTAAGTCGTTGAGAGGAGTGCACTTT
CATTATGCCCATCCCTCACTTCAATTGCAACGCATTCTATACTTTCCGATTGATTAGGCCGATGGACAAG
CGAGTCGTATTAGCTCTTATGAAGCTGTCATAATCAAGTTACGTCTCTGAACTGCCATTCCATTGACTAA
TAGACAGAAATGGTTACAAAATTATGCACACGTAATATTTATCCTATGGATTATAATGAAAAATTCATC
AATTTGAACCGATTGCATATTGTGGACAACAATGTAACCCATTAGTGTTGAGGAACCGATACCCTTCTC
AAAATGCAAATTTCCTGTGACGTAGATATAACATCCAGTACCCTCCTAGTTAAAGGAAACCATTTGCTG
CGCAAAGTTCTTACATTTAGTATGATACCGTATGCCCTTATGTGTAATTTCACAGATGGTGTGGAATCAT
ATGGCATGGTAGTCCATTGATAAAGAGACCTAGGGTTGATATCTCCACCTATTGGGACATTCATTAAGA
GGATTGTATTTCGTCTCTAAAAACTTATGAGTTTGTCGTCCCTTGGACGTGTGCGTCATATTGGTAGGAA
CTATCAAACGTGTCTCCTCGTTGGTACGGTTCATGTGGTGAAGAAGTGAGTCCCACAGACATGGAAGCG
AGTAAAAACAAATGGCAAAATGACAACAACTCTCGGAGACAGTGTGTAGTAGCATGTAGCTAATATAT
TTGCAAGTGTTAGTTGGAGAGGCCAATTTCATGAAGGTTTAGTGGCAGCCTATGAATATCGCACTTTAA
TTTGATAATGGCATGGACCAGAATTTTCTAATCTATTTAGTTGCGAGGAGTGGACTTCTATTATGTACCA
TCACTAGTTTTATTAATGACGTACTCTGTCCTTTCAAATATATTAGGACAACGAGCAAGCGAGTCACGTT
TGAGTTTATTTGATCGCTATCATCAAGTCATGTTGCTTAGCTGCCATTTCGTCGACTAATAGAGGGAAAT
TAATTACACAAGGATGAATGCGTGTATTCCAAAATTTACTATGATGATTGAAATAATTATCCAATGAAT
TATCACCACAAATTCATTACTCTACATGGATCGTCTGTTGTGGACAACAAGGTAATCCATTATGCTCGA
GAAATGAAGAACCATATCAAAATGCAAAATCCCCGTGATGCAGATGTAACATCAATACTCTCGTCCTA
AAATCGTCCGTCGTGGAATAAGAATCTAATCTCCAATGTTGGTACATACTAATTTAGCTTGCAACAAGT
TATTGCATTTAGTTCGCTTGACTTGATATGGGGAAGCAAGTCTATTTACGTGTTGATTTGGTCAGGGTTT
CTCGGTCATCATCATCATTGATATAGGGTTCGTGGAAGCACATGTACGAATAAATATAAAAATGGCGAC
ATTTGATAGATCATTATATGTATGCCATCACATGTAATTTGAGACCTAAAGTTCATATCCACACCAGAG
AGAACAGGGTGACACGTCTCCAACGTATCTATATTCATATATTGTTATATGCTATCAATATTTAATACTT
CATATGCTTTATATGCAACTTTAAATTATTTTGGGAACAAACCTATATTTAGTAGTTGGAGTGCACTTTC
TATATTTTAATCAACAACATAATCATTTTCATCATGGTCTGAACTCAGGGAATTTTTTAATCAAAGCGAC
AATCTATATTTGATAATAAGCAAGTAACATCAAAATGTGTAAACAAAAATCACTATTGATTTCAAGGA
ATAATGTTTTTATGTAATACCAATCAATACTCCTATTTCATATCATGTCCCCAATCAATCGAGGATTTGT
CATATTCAACATTTTATCATACACAACCTAGAGTTTCAGTCAAACAATTAATATTTATGATGAACCTTTT
CAACTACTTAGCACTTTGTTCAATGATGAACTTACAAGAAGTGGTCTTAGCACTTATGATGGCAAATAG
AATGATGCTGGGGATTTATAAGAAGACAAAAATAAGAACGACTGTCATCAATGC

>TaeSat04-337
ACGAGTTTCCGTATGAAACCCTGATACTTTGAAAGAGATTGTCCGTTTTGTACACGAAGTGCATCCAGT
TTTTGCCGTAACCCTCTCTACTTTCTTGCACATGCTATGTGGGTGAAATGATGATACCATGCCAACTTTC
AACCTTTTCAGAGTTCATTTGAAATGCTTTTCAATTTTAGGGTCTTATAGCTCAAAATAATTAGTAAATG
CATGAAAAATAACAAATGAAGTCAGAAAGGATTGAAAAATGATGATGTGGCTTTGAATGGTGCATTTT
GAACACACAAAAAGTCAGGAGTTCAAATAAGTTTAAAAAAATGAAATCCCTTTGTAACAG

>TaeSat05-500
ACGTGTCCCCTTGTGGGCCAAACGTAATCACTGCCTCTTTTCGCCTTGTATTTTCTTCTACGGGCAACAT
GGAACGACAGGATATCCGTGCTGAAATTTAAACTTATTCAGGGTTCGTTTGGCCTTTTTATACACTAATT
GAGTTTCCTAGGCATTTAATGTCCATAATTCAAATCTGAACTACAAATACATGCTCCAGTTCACCAAAA
TGGCTAGAAAAATTATACATGTGTCCTTGGGTGCATGTTTAGGTCCCATGCCAGGAATGGGAACGAATT
ACAACCGTACCGGTGTCCTGGCTCGTCCTCAAACATTTCGAATCTCGGTTTTTAAGTCCCCATAAATCCT
AAACTCACCAGAAAGTCATCAAAGGTGGCATGGTGTCACGTCATGGCACATATATGTCGTGGTAAAAA
CATTGCCAATTTGGGCCAAGCTTTATTACAAACCTCTTACAAACCGGAGCCTGTCGCAAGAACCCTCGT
GGTTTCGATAGGGAA

>TaeSat06-403
CTTTATTTCTGTTTTCAGCATGTCCCAGTTTTTCACTAAGTCTGAAAACTGATTATGATTTTGCTATGTTT
ACATGCTTGCTATCGAGTGATCTGAGCCTGTTTTGCGGATGGTCAGTAAGGGACTTTTGTTAAGCTTGTT
GTGCTCTATCCATCCATGTCTTTGTTTGCAATGATGGGGTGCTGTAGCATGATTCAATCGAGCTCTACTT
TTGCTATAAAATGTTCCTGGCAGATTGTTAACATGTTATTCAATTTTGCCAAGGTTGTTGTAGTTGATCC
ATGCATGCTATGCACTTGTTCTTGCCATGTTTAGCTTCAATAACATGTCTTCTTGATGGTTGTATGCTTAG
CTTGTCATGCATTGCTCTGTAGTGAGTGCATCGAGCTCGTAAACATGCCTA

>TaeSat07-343
ATGAGTTCTCGTCCGAAACCCTGATACTCCGAAAGAGATTGTCCAGTTTGTACACGAGGTGCGTCCAGT
TTTCGCCGTGACCCTCTCTACTCTTTTGCACATGCTATGCGGGTGAAATGATGATACCATGCCAAGTTCC
AACATTTTCAGAGTTCATTTTGTAGTGATTTTCAATTTCACGGTCATTTAGCTCTCTAAACAAATCGGTA
AATGACTGAAAAACAGCAAATGATGTCAGAACGTGTTGGAAATTGATGACGTCGCTTTGAATGATGCA
TACTGAACGCAAAAATAGGCTGGAGTTCAAATAAGTTTAAAAAACATTGAAGTGCCCGTGTAACAG

>TaeSat08-663
GGACGAAAAATTCATTTTCCATTTTTCGAGTGCCCGAAATGAGGTTTTTTTGTGAAGGACCTACCAAAT
AATTGTTGCAAAATTGGACCAAATCAATTTTATAAAATACTAGGCCATATTTAATGCACAATTGACAAA
ATGGTTGGGTGTAAAAAGTTTTGATCCACCTCTCGTGAAAAAGACAAATTTCCGCCGATTCAGTTGGAA
GCGGGTCAAATTTGAACTGCAGCTGCCTCATAGTTTGCTCTTTATTTTTTCCAAAAATCATTTCTAGGTA
CATAAGTATCTATTTAATCAGAGAAACACCAAAAAAAATTCCAAGATTCAACCACTAGCTAGGAACGGT
CATGCCCGCCGTTTTGACCGCATTTTGAAACGGGCATAAAAAAATTCAAAAAAAATCAAAAAATTGGAA
AACCTTCGCATTGTGTCATTATATGTGACCAAGTTTCCAGGAAAAATAATAAACTTGTAATACGGCAAT
TATTTTAAAAAAGTGTTCTCAGAAATGAGCTATCATGTGTGAAGATTCATGGCTTTCAAGCCAAATGAT
CAATCTTATGGCCACATTCATGGCATAGTTTGTTCAAATGATCTCATATTGTGCACAAGGGTGCATATTG
GAATGGCAAACAATGTTGCCTAAGGAAGTTTTCATTTTCTTT

>TaeSat09-335
GTCTCCGGGGTGTGGCCCCCCTCACGGACGGCGCCGCCGCCGGCACCTGGAGGGGGGAAACGGACGCG
TGGTAACTACACGGAGGGGATCTTGCTGTGGGTCTGGAGCGCGTGTTGCTCCAAAGTGTTCCTATGGGC
TGACCTAACACAACCGGGTGGAGAGGTTTTTACTGGTCAAAGCCCATCCGTGCAAAGTCAAAGGGCTA
GATCCCGTGGTCAAACGCTACAGGGTTAGGCGGGACGGGGGCCTCGGGGGGCTAGAATGCCACCGGAG
CGTCGGTAGGCGCCCTCATACATGCGGGGTGGTGTTGTGGCATGTCCGAAGAGGCGGCACGC

>TaeSat10-206
ACGAGTTTCCGTATGAAATCCTGATACTTTGAAAGAGATTGTCCGTTTTGTACACGAAGTGCATCCAGT
TTTTGCCGTAACCCTCTCAACTTTCTTGCACATGCTATGTGGATGAAATGATGATGTGGCTTTGAATGGT
GCATTTTGAACACACAAAAAGTCAGGAGTTCAAATAAGTTTAAAAAATGAAATCCCTTTGTAACAG

>TaeSat11-506
CAATGCATGTTTGATGACGAACGGGAGATAGCTTCCTCTTACGGCCTTCAATTTTTTTTCTACGTTTAAC
GTGCACTAATACAACTGTCATGTGAAAATTTGGAAAATTTCAGGGGTCATTTGACCTTTTAAAGACATT
TAAGTGATTTTCTAACCATTTAATGACCGTAATTCAAATTTGAACTACATCTACATGCAACGCCTAACCA
TAACGGTTTGAAAAATCATATTTTTGTGTACTTGTGTGCGAGTTAATTCCATGTGCAGTAAATTAGAAG
GAATTTTCAAACATATTGGTCTCAAGGCATGGGCACATGCATGGAGTGCCATGGCATTTTAATTCCAAA
AATTAAAAAATTATCAGAAAAAACATGAAACCTTCCTTGATGTAATGTCATGCCACCAAGATGATGTGG
TAAAAAATTGGCATGCTTGATGAAAGTTTGGACACACACCCCTCACAAACCGGAGCAACTCACTAGAA
GGTTCGTGGTTCCGAGAGGGAA

>TaeSat12-369
CTTCAAAGGATTCTTTCATAGGGAACTTTTGGAGAACTTTTCTTAAGGAGTTCGTTCAGAAACTTGTATA
GAAACTTTCTTAATGAGCACTCGACAAACGCATTTCGAGAACATACACAAAGTCCCAGCTATGGACCTA
GTGCATCATCATAACTGAGGTCTCTTGCATTTCCCTACAAGTAATTGGATCTCATATGGAGATGTTTAGT
GAAGTAGAATGGAAAAAATCGAATGCAACTACACAAGAACTAGTCTGTAACTGTCGTTCGGAATCAGT
TTTGCGCAGTGTTAACTGAAACATGGATATCTGACTCATACGGAGTCCGTTTTGGCTCCACAAGTAGTC
AAAACGTTTGTGACGACCAGATG

>TaeSat13-44
GAACTAGCTCTATAAGCTAGTACAAATAATTTACACTAGAGTT

>TaeSat14-1463
ATTCAATACACATTTTTTTGCATATATGACAAGTTAATGTTTTGACCTTCAAGTCATCTAATAAATTTTA
CACATGCTCACTTACATGTAATACACATGGATATTAATTGGATTTCAACAAAAATGAGGCCGTGGTGTA
TGAATTCTACTACCACATGCATGCCATGGTCATGGTAGGGTGTGCATAAAGTTTGGGATCATTTGGTGG
GACCGTCAAGGAAAACCAATTTCAGACCTGATCTCCGGCAGACCCGAATGGTCCTGCTTGTACATGGTG
CATGTGGAGGCATGCATGAGATGACCCCAACTTTTGGGACCATGTGGGCATGGCCAATGTGGTCCCCCA
GGCAAGGTGTGCACGAATTCGGACACAAAACGCACGTTGCGTCACGTGGGGGCAGTGTTGTAGGGTTT
TGTCGCCGGAAAAACCCTAGAAAATGCATCGAGTGTCGGAAATGAACGATACTTGTCATGCATGCTTGC
CATGATCATCCTTGGGTATGCATACAGTTTGGTCTGAATTGGTGAGACCGGGAAGGTAAACCTGCTTCT
AGTACATGGTGCATGTGGAGGTATGCATGCGACTGTCCCAACTAGATTTGATTTAATAATATTTCAAGT
ATCAAACAGAAAATAAAATTTATAATTTGGAATGAAGAATTGAAATAATGAAGAAGTGCAATAGAACA
TAACATATTATCTTACATTTTGGAAATATTTCAAATATAAAGAAGAGAACAAATATTAGAATGAAGAAG
TGAAATAAAACAGAACATTTTATCTTACAAACTAAAAATATTTCAAAAATAATAAAGATAAGAAATAT
TAGAATGAAGAATTGAAATAAAACACACCATTTTAGTTTATATTTCGTAAATATTATCAATATACAAAA
GAGAACGAACAAATGAAATAAAACACAACAGAATTTGGCATATTTTTTAAATTTTAGTAAACACTGTTA
GAAATAATATAGAAAAAAGAGATTTGATTTTAAATATTAGAAATATAAACAAGAAAAGAAACAAAGA
AAATTTAAAACGTATCAGCGAGCGGAGTAAGGCTGCCCTGGGCCAGCCCGCCCGAATTGGGCCCAAGG
CGGAGCCGCGCAGGGCACATCGCAGCGCATAGCCGTTGGGTGGTGGGAGTTTAGTCCCACCTCGCCAA
GCGAGAGAGCGCCGCACCGGTTTATATACCCGGCTGCGACTCCCCTCACAAGCTCCTATCAATTGCAGG
CAGTACATTGCCTAACTCTCGTCCCCTCTGCCCCGTGGGGCCTACTAGCAGCAGAGATATTCTGCACCA
CGGGCCTGCATCCTGGCCCATGCACCGCAGGGTTCCTAGTCGTATGCAGGCCGTGGAGTATGAATTCTC
CTGCTCTGGTAGGTGGGCACTTTTTTTGGCATATTGCCATGTGTATTGATCTTCAAATCACACACTAAAT
TATACACATGCTCACTTGC

>TaeSat15-620
ATAATTTGGGCATATGAAGGAACTGTATAAATTTCATGTCATTTAGAGATATAAAAAAGGTACTTCCTT
CACAATGCTTCTAGGTGGACAAAAACTTTGGAAATTTGCCGAGGAAGATTTGCTAGGCAAATGGAGCT
GAATTTTGTCATGCGGTAATGATTTGGATAGGAAAGAGTGCCCAAAAATTCCGAGGGCAATCAAGAAT
ATATAAATAGCACTTCCTTCACAAACTGAAAATATGAACAGAAACTTCGGAAAATTAGTGAGAGAAAT
TGGATAAATGAATTGAGCTCAAATTTGGTGTAGGTAAGTTACATAGGTATGGTCATGCGCTGGTAAATT
TTCAGATCATTTGGGTAAGCCTAGCTAGTACTTACTTCACAAAGCTTCTCTCGAGGTAGAAACTTTGGA
AATTTCCCGAGAAAGATTTACTAGGAAAATTGAGCTGAATATTATCATGTGGCAATGATTTGGGTATGG
AAGAGTGCCCGAAAAGTTTGAGGGTAATAGGAGGGGTCTATATAACACTTGCTTTGCAACGTGCCAATT
TGGCCATAAAATATAAATTGAACCTGGGCTCACATAGATGATTTGACTGAGCTGCAATTTGGAGGAGG
GTG

>TaeSat16-567
CTCGTGCGCTTTTTGCCTGATTGCCCCAAAATTTCTTGTCCTCTATAGAGGAGATATCGTTTCCACATAT
ATATTGACCGATGCCAGGAATGCCCTTTTGTTCTTGGCTTTCACGCGCATGAGTTACAGCATGCCATCCG
GCGAAAACAAGGAGAATAAGGCATATAGCCGCTGGATTTTCTTCAATTTACGGCCAAATGAGATTGCC
CGGATCATAGAGCATCTGCAGAAAGGTACGTTTGTCCCTACCAGGGGGGCTAATGTCTATAAGCAACAT
AATCTCTCGAATTTCCTTCAAAAGGAAGTGCCAATGGGTTCAGAAACTTGTCTGAAAAACAGCGACGA
ATGATGTTCTTTGACTTTCAGGGCCAAAAAGTCCTGCTTAAGGCCGAATTTGAGGGTACTCCGGGCCTC
CTGGCGCAAGCGGGGAGTATTTTTTGGATTCGCGCGCTCGCCGCGAGCAGTTTGAGTACTTGTGGGTCC
GACACGGACTCCGAATGACAAAATTATGACCGTTTAGTGAACATCTGTCCATTCTATGGCAGTCCTTG
AAAGTACTTCATTTT

>TaeSat17-323
AAAATATAAAACCTTGGAAACCTAGCTTTGTTTGTGCAAGAGATCATGTGTGCCAAAATTGAGGTGATT
TGGAGGTGGTCGAAAAAATGACCGCATTCCGGAGGGGCCATTTGGTCTAACTTAAGCCAGTTTTTGAAA
AAAGGTGATTTTTTCCACCACCTCCAAATCACCCAAATTTTCTTGTACATGGTGACCCACATGTGCCAAA
CATGGCTATGAAAGAAAATTTGGAAAAAGAATATTTTACAATGCCCCCTTGAGGTATAGACCGATGTTT
TGACCAGTCAGTCTAGAGGGCATTATAAATTAATAAAAAATTCAAA

>TaeSat18-733
TCAAACTATCAGATTTCTATAGGTAAGTTGCTATCTGAGGTGCATATAGATGCATGGACTACCCTCGAA
GCATGAAATGGTGGAAACGATTTCGGAATATGTCGATGACGCTTAATTAATAAGTTTTCAGTTTTGAAC
AAAGTGAAAATCTATAGTGATGGATCAAAAGGGAACAAAATTGGTGATGAAATTATATGATTACTAGT
TATAAGTTGTTATACGAGATCCATATGGTCGAACATACAACTTCCGAAGCTTGAAAGGAAGAAATGCTG
GTGGAATGTCTAAATGATGCTATTTAAGTAAGTTTTTTGTTTAGCCAATAAAAAATCATCGGCAACAGA
TCAAAAGTTTATGTAAAGGTGCTAAACATGAATAATTTATGTGGATAAGCTGTTCTATGACTTCCACAT
GGCCGTCGAGACGACTCTCGAAGAATGGAATGATGGAAAGGCTATTATAATGTGTCTAATGTCCTATTT
AAATGATTTCTGAGTTTCAAAGGAGGAGAAAATGTAAAGTAATAAATCAAAAGGTTATGAAAAACTGC
TAAAAATTATGTGACTCCTACATATATAAGTTGTTGTAGGATTTTCAAATGGCGGCATAGACAACTTCG
AAACAAGAAACGAAGCAAGGCTTCTATCACTTGTCGATAATGCTATTTAAACAAGTTTTCAGTTTCGCA
AAAATAGAAAATCTACCATGATGGATCAAAACGGTTAAAAATGTA

>TaeSat19-653
TTTTTCATTTTTTGAATGCTAAAGACATGCGTTTTTCGTGAAGCGGCTACAAGGAGGGTCACCCCAAAC
GGCGCCATTCCATGTCTATCTCAAAGTAGACCCTATTTTACGGACGGTCGCCAAAAAGCATGCATTTCC
GACCCTCGTAGCTACTCCCGGCAATTCAGACCACCTTCGGCCGATTCAGCTGGAACCGGCTGGAATTTG
AACTGCGGGTCCTCCATAGCTTGCCCGTTATTTTTGCTAAAAATGCTTTTTAGCTTCACAGGTAGGCATT
TCATCACAGAATCGACAACAGATTGGCACGGCTCAAACCCTAGCCACGGACGGCCGCCGCGACGAAAT
CGGCCGGTTTTTGAAAACACCATAAAAAATTCAAAAAAATGGGAGACCTCCGCGTCACATCATCAAAT
GTGGCCTACCAACTAGCAAAAATACTAAACTTGCAATACGGATGTTTTCTTGAAAAAGTGTTCTCAAAA
ACGACCTACCATGAACGAAGATTCATGGCTTTCAAGCCAAACTAGCAATGATATGGCCGCATCCGTTGA
ATAGTTTCTGATAATATGCCCAAATTTGGCGCATGCCTCCGTCTTGTGATGGCAAACAATGTTGCCAAA
GCGAGGTTCCAACTTGTTTAACAAGAAAAACCG

>TaeSat20-322
CTCCGTGGTGTGGCCCCCCTCCACGGAAGGCAGAGCAGCCGTCACTTAGAGGGGGGATGGTGCGGGTG
CGGTGGAACCGGAGGGAATCTAGTGTTGGGTTGGGTCCAGACCGTGTTCGGGGATGTTGCTATGGGCTG
ACCTATCGCAACCAGGTGGAAGAGTCCACTGGTCAAAGCCCGTCCGGCAAAAGTCAAAGGGCTAGATC
TCCTGGTCAACTGGGATTCGGGGTGGCCTTCGGGGTGTAGCTATGCCACCGAAACATCGCACGGGTCCT
GATGCATGTGTGAAAGTGTTCTGGCATGCGTAGACGCCCGTCTTGGGG

>TaeSat21-1590
CACCAGGCGGCCTTCGCCGCGCTCGCCGGGCCGCTGCAGCTGCCATCCATCGCCACCACCGCCCAGCCC
TGGCTGCAGTGGCAGCCGCCGCTCCTGGCGGCCTCCGCCGCGCCCGGCGCTCCGCCGCAGCAGCCGTTG
CCGCTGCCCGGCGTGACACCGCAGCAGCCGCTGCAGCTGCAGCAGCCACCGCCGGTCAGCTCCGCCGC
CCAGTATGGGATGCCCTACGACGGGACTGCGACGACCTCGTTCCCATCAGCGCCGCCGCCATCCCAGGG
CGTCCACATCCAGCAGATCAAGTCCCCGCCGTCGCCGTCACCGCTTCCGTCTTGGATCGCTACCCGCCA
CGTGTCGGCGGCGGTGAGGCTGCAGGCTGCTGCGCGCGGCCTCCTAGCGCGTCGGCGTGTGCGGGAGA
TGCGTGGTCTGCAGCTGCCGCTCCTCCAAGTTGCCCTTCGCTGCGCAAAGGACCTCGATCTCGTCCGCTG
CGTCGGGGATCTTGGGCATGCGGTTTCCCCCACGGGCGGCGGGCATGCTGTTTTCCCCGCGGGCAGCGA
CCTCAAAGTCTGCGACATCGGCGGTTGGGGGGGCGCACCCCTCCTCGTCATTCTCCATCGCAAGCCCTC
CACTCTTCCCTGTGCGGTGCAGACCAACAGCCGTCCGGCGGGGAGAAGGCATGGTGTCACCGACAGCA
GCGCACCGCGTAGCACCACTGCATTCCGCCACCGGCCGCCGCGAGGGCGCCTCTGCTGGTCACTCTTGC
GACCACTTCCAGGTGGCCATACACATGCACTCCTTTTGTCCAGGTGGTGTCCATGGGATCCAGGTGGCT
GTACACGTGCACGTCCGACGTGCGGATGGTGTCCACTTTTTGTTAAGGGGTCCAAAATAAAGCGTCCCA
GTCCATTTCAGGTTGAGAGTAATAAAACAAGCCGAGATGTAAAAGGCTTGTTTTTAGGTGTTAGGTTTG
TGTTGCGTCGAGTCATGGTTATAAGTTGGTTAGGCTGCAGCTCGAGGACAAGCTGCATGTCCAGGTGGG
GTGTAGTGTTAGAGTACGTAATGGGCCTAATGGGCCCATTAGTCTTAGGGTTAATTAGAGATAAGGGTC
GCTTGCTTAGGGGTCAAGTAAGCCTTGCTTGGGAGTCAAGTAAACCTCTCTATATAAAGAGAGGAGATG
TATCAATCTAATCAAGCAAGAATTAAGAAGGAAATCCCTTCCCTCTTGCCCGGCCGTGGGCAAAAAGG
CCCCCGGCCGGCCCTCTCGCGCCCTCCTTCTAGCAGCGCCATAACAATTTGGTATCAGCTAGCTTCGGTT
CGATCATGTCTTCACCGCCGCCAAGCCCGTCTCTTCCGCTGCCGGTCACCTTGTCGCCTCCGGCGACCAC
CACGACCGTCGCCCCGCTCCTGCCCGCGCCGGGATCCTCCGTCGCGCCCGCCCCCGCCGTCCTCACCCC
GGAGGAGGTGTCCGGGGTGCTGCGGGACCTAACCCAGGCGGTCCAGGAGATCCACCTGTTCTTGGCCG

GGTCCTACGGGCCGCACCCGGCTGCGCCGCCCATCACCGCCACCGCGCCGCCGTGGCTGCCGTGGCAGC
CGCCG

>TaeSat22-320
TTGGGGTCCACCGGCTGAGTCAGAAAAGAAAACGCAGTACGGGGCTGACATCCTTCATATCCGATCAG
TGCCGGTTGCATGGGTGGTACGAGGTGGCATGCATGAAACGTCACCCAATTTTGGGAGGCAGTGGGCA
TGCCCATCTTAGGGCCCCACGCAAGATTTGAACGAATTCGGACACCAAACGCACGTTGCGTCACGCCCG
GTCAGTGTTTTCGGTGCGTTTCACGGAGAAAACCATAAGAAATTCATACGGTGTCGGAAAATTATGAAA
ACTTGCACGCATGATGGCCCTGGTGATGAGTGCGTGTGGAAAAAGT

>TaeSat23-319
GAAAAAAATGATCGACTTTGGCGGATGCGACCGGGAGAAGTACGACCGAGGGTCCCCTCCGCCCATAC
CGAAACCACCTGGTTGCACAGCAAGTGCCAGTTCGGTTTGCCGGAATGCACCCAACTTTTGGCAGCGCG
TGTGGGGCCCCGCCCGGGGACCGCACGCCAGAGATGAACGAAAACGGACTACGAACGCACGTTGCGTC
ACGTGCGGGCAGTGTTTTAGGGGTTTTCGGTGCGAAAAATCATAGAAAATCCGTACAGCGTCGGAACG
CGACAAATTTTGGCGTGCGTGGCCTGCATGGTCACTGTTGCCCGTG

>TaeSat24-338
CCGGGGCCACCGGATGGGTGCTCGATATAGAGACCGCCCGAGGGGGGGAGGGCACCCCTACATGCGTG
TGGCCCATGCATATGCATGCAGGGGTGGTGTGCTATTTGAATAAGCAACGCACGTCCTTGACGTGGCAC
GTGCCTCACAAACCACACACACGGGCGGGAACGTCGAGGACCGGCTGCGTTTGTCCCCAAAGACAGAG
TCTTCGATGTGATCCCGTGACAGAACGTTCCTAGACTTGGTCTGTCATCTCGCGATGACATGCACTAAC
ACGGAGAAGCAGTTGTTGACCGTGGCCTACCCCCTCTCTCGGTGCCGGTCAACTTCCTAGACCG

>TaeSat25-318
GTAGTTGGGAACCCCCTCCCCGGCAGACCGACAAGTAGCTGGTTGCACTGGGGCTACGTGATCGCATGC
ATGCAACTGCACCAAAGTGTGCGTACATGTGGGCACGGCCAACGTAGGCCCCCACGCAAGGTTGGAAC
GAAAACGGACACAAAACGGACGTTGCGTCACGTCCGGGCAGTGTTTTTGCGATTTTGGACCTGGAAAA
CCCTAGAAAATGCGTGGAGCGTCGGAAAAATACGCGAGTTGGCGTGGGTGCTGTGGATGGTGATGGCA
ACGTGTGGAAAAAGTGTCGTGACGTTTGACGGAGAAGAAAAAAAA

>TaeSat26-210
TTCCTTTAGTTTGCTCATTCGCTTAATTAGTTACATCGAAGATCTAGACAGGAGGTCCTGTGTTCAATTC
CCACAATGTTGATTTATTTTGACCCAATTATTTTTCGCGGTCTCTATGAAAGCCCATAAAAGGCCCATCA
ACATAGAAGTACCTGGCCCAGATTGACCAAACGAAAAGGACTAAACCAAACCAAGAAAAGGACGCAA
TCC

>TaeSat27-72
TCAATCTCAAGATGTTGTGTCGGCTGAGTCTCTTGAAGGTGATCCACCCAGAAGGCGTATGTGGTGACT
TCG

>TaeSat28-543
TCCGAAACTCAATTTTACAAGCCGGATCTTGCCTCCGAAATGTTGTCGAAGCCCGCGAGTGGGTTACGG
GCGCATACAACTTTTGGTTGTGATTGTTTTGGCGGGTCATGGAGCTCCAATGGAGTTTTTATGGCAAAAT
TGTGGCCGTTTTATGGATACAACATCGCGGGACAGACCGTGAATACAACTTTCAAGGTAAGTTGATCGC
ACCGACGAGCCATCTTGCACCATTCGGAATGACCTATAATTTTTCGAGTGCATAAACGGAGTGAGGATG
AACTGTTATGTACTTTATGATCCAAGAATAATGCATCCGCTGGTGAAAACGTGAGGGTGGTAGCGGGAT
GGGTGATAGACCATGTAAAGCATGAATTCTTGGGTTTCGATGCAACGAAGAGCCTCCTCCTTGTGTCGT
GACTGAACCTGTAGTCTTTCTAGTTTGTAATGGAAGGATGGTGCATTGTTCTATATGTTATTGTCCATAT
ATCAGTCCGTAGGTGAGCTCACGGAAGGGTGGTAGAGTATACTTTAAACATAAAATCA

>TaeSat29-319
CCGTGGCATCATGTTGCGTGTCAAAAAAACATGGCGGCGTTTCATGGCTTTGCGAGAGGGAACACCTAC
TTCAGGCATGTAACCCCTGACCGACACCTCAGGTTTGTACTGTGACTATGTGCCAACATGGACGAAACG
CACCCAACTTTTGCGTGCGCGTGTGAGTGACCCCCACCGGCTCGCACGCCTATTTTCGACGGAATCGGA
GTACGAACGCAAGTTGCGTCACGTCGGGAGACCTTTTCTGGGTATTTCCACGGAGAAAAACGTAGAAA
ATGCATCGACTGTCGAAACGCACCCCAATTTTGCAGGCGTGCTT

>TaeSat30-1389
AGTAGTTCCCAGCTAGCAACTTTGCATGAGTTATCATTTTGTCCTTTTTCCTCTGCCTTCTTGCCAAGCTC
CAGTATCATATTGGCAGGTGAAGACCACCTCGCATCATGTGCGGTCGGATGAGGATCGCCAGTGATGCT
CATCTCGAAAGTGGTTTATAGAGCTGACCACTTGAAAATTCTTTTGCCCAAGCATGCTAAGCATAGTAC

ACAATGTACGCTAACAGTTCAATGCATGATACTAAGGGGATACTATAGGGATTGGATTCAATAAACGCT
AATCAGGAAGTACGTGGCACAAGACCCATTTGTATGAAATTCTTTATTATTGTGCCTATATGGTCCATG
AACCCAATGCTTGTCCATTTGGAAAATTAAAGAGATAAGAGTAAATGTACTTACACATACCATCTGCTA
GTGAAGGTACTACTGTGTATCTAGGGGCTATCCAATCACAATCACAGCAGATCTCATCAAGCTCCTGCA
TTGACAAGTGTCATAGATACATATTGCATTTAAAAATTTAATGCATCCGTTTAGCACAAGGTTTCTTAAC
ATGCATTTATCGCGTTGGGAAAGTGCCCCTTCAAGTTCAATCCCATCATGTTAGTGCTCTTGGTTTTGCC
ATCATCGCCTGAGAAAGTTAGATTGGCAGCTGTCATTATATTTGAGCTAGGTTCCTTGTATTTCTCCATT
ATTGACAACACTTTTGCTGTCATCTTCTCTTCTGGATAAGGTTTAAACATGATGAGTAATGGAAATGTGG
AACTCATATAGAAATAACTTGCCAAAATTTGCCACACTTCTAGTGAACTACAAAAAAACAAATTCATAA
CTTTGATAATGGTGAATAGTACATGCATTATTCATTATTAAAAATTCACAAAAATTTCATTTTTGTGTAG
CTCACATGTTAACATATTTCATCGTGAAACATTATACACTTGTGGAATGCATCCATATGAACATATGTAT
AAAAACCTATGATTTTTTTCAAACTTTGAACTATGGTTTTTGATATTTTTAAAATTCAAGCTCCAAAATG
CCTCACTCGCAAAACCTATCAAGATAAACAGGAATGCAATTGCTAAATTAGCAATAATAAGCAACCTTC
CAAAAAGTGCTACAATTTTAGGAAAATGTAACCCACTCAGATGCACATAAGTGAAATTCCACCCAAAA
GCAAGTTTGAACAAGTTCCCCCCAAAAAGGAAAACTCAATAAAATAATGGTAAAAATAATTCGTTTGA
GCACCTGGTTAGCACGCACGTGCACAACGCAACACAGTACAAACTCATGCTACACAAATCATTATAACT
TTCATACATGAGAACAACAAATAGCTAAGTTGCTTTATTTCACTCTTTACGAATATTGGCCCCAAAGGC
CT

>TaeSat31-889
CTGTAAATCAAATTTGCTGATCCACGTGTTGCAAGAAGCCTTGCAACCTATGGCAGAAATACACACAAT
AATTAAGACAGCCAGCAGAAACAACAGTATTCAGCGACCACATGAATACAATGCCTTGGCGTAGCTAC
TAAGCATCAGGTCAAGTAAATAGGCAACAAGCCTTGTGTTCCATTGGATTGCATCTAAAGAGTCCTTAA
CTCCAAAGATTCGAAGAGAAAACAATCAAAATCATGCCTGAAACAAAACACGCAAGAAAAAAGTAAA
CATGTACCCTGGAGAGGGCGAGCCGGCAAGCCATGAAGGGAATCGCCTGGATTTTTACTCTCGAGGCT
CAGCGATTCGTAAGGTCTGCTTCTCCCTGTCTCGCGATAATAATCTCTACTAGAGAGAGAGAGAGACTA
ACTATAGCCGGAGAGGCCGATGCATAACTGCGGATGCAACACATAATTGGCATCTCTTGGTTGCACCAT
GGTGTAAGCATTTCCCAAATAGGTGTTGGTGACATTTAAAACATAATAAATCAGTAATACCACATAATT
TGAAGATCGTGATTTACAACAGAAATTGCAAGCTCATGTATACCGAAAAATATAAATGCTTATGGTTGC
AATAGAATTAGAAATAACCGAGAGCCTTAAAAAAGGCATGTTGAAAGTGATAACTTGTGATTCCATTG
TAGATGTCAAAGTACATCGTCCATGGATGGAGCAGTTAACCTTGCAACCCCCAACAACTATCTGCCTGC
ACTGTAATATTCTAAGACGATGAAATTCCCATTGAACAATATATACCTGCAATAGTAGATTCTTTTTTTG
CAGGAACCTCAGTAGTAGATTCAGCATGTAGCCTGAAAGAAGGAACATAAGACGTCGCTGTCTTG

>TaeSat32-528
GAAAGGGTACAAACAAAATTACTAGTACAAACAAAGGAGTATCACTATAGATAGATGTTGCCGGCCCT
AGCTGCATTGATCATAGCTGGTAAATTCAAAATAATTTCTTCAGTTATGATGTAGCATTGATAGATGCC
AGGGTGGCTCTCCAGGTTCGGCATTGCTTTGAAGCAACACCACTGATTTGTTTGGCTAGAGCACACTTT
CTTGTCAAGAAACCGAATGAACTCCACCTTAAATCATATATTTGGCAGATCTTGTGAAAAATCAAAATC
TAAATGGGTAACCATTAATATAACAGAGAGCGTGCACTAAAAGAGGGCCGGGGGAAGCGGAAAGCGT
TACAACATTCCACCTTCAGAAGGGTTACAAATGGGCGGCCTTTGCCCGTTTCCAAACTTGCGAATCCGC
GAAATCACCCCGCTGCGTGCCGCCTCTAGTATCAGGTTGAAAAAAGATCCGTGTACACGGAGGGAGGA
AGTAGAACGGACCCGGTTCCAAACATCCATTTTTACGGTTGAAAAAAAA

>TaeSat33-54
TAAAATACAGTATTGCGATTATCCACCTCAATTTTATATCACTAATTTCAGTAC

>TaeSat34-175
CAAGTTTTTAATTCACGCAAATCTAGTTCACCAAGTTCAAATTCGGTTCATCAAATCATAATTTTAGTTC
ACTAGATTCACATTCAGTTCACCACAATTCTCATCATAATCTTAAAGTTTAGTTCACTAAAAACACTATC
TAATTCAGTTCATTAAAATATGTTCACCTGGATT

Supplementary Figure S2. Repeat landscapes plots for satDNA families in bread wheat (T. aestivum L.). For each satDNA the abundance (Y axis) and the divergence (X axis) with respect to the consensus sequence was built according to the satDNA repeat unit.

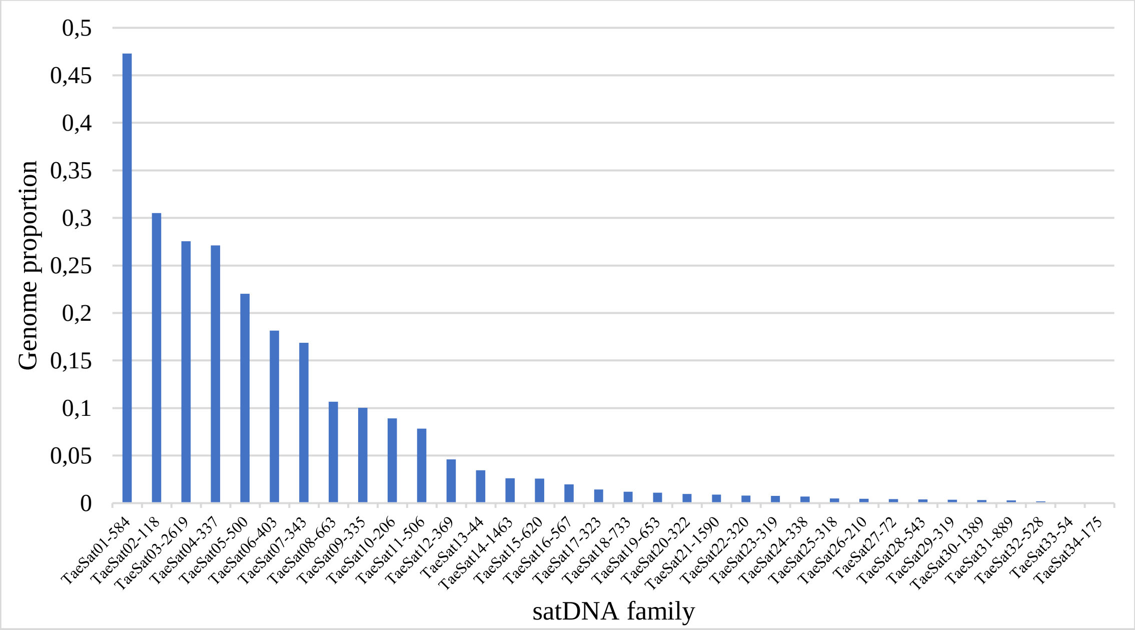

Supplementary Figure S3. Secondary structure prediction for satDNA Taesat21-1590 dG= -301.78; TaeSat14-1463 dG= -170.87; TaeSat01-584 dG= -97.28; TaeSat16-567 dG=-60.15; TaeSat28-543 dG=-55.27; TaeSat12-369 dG=-34.59.

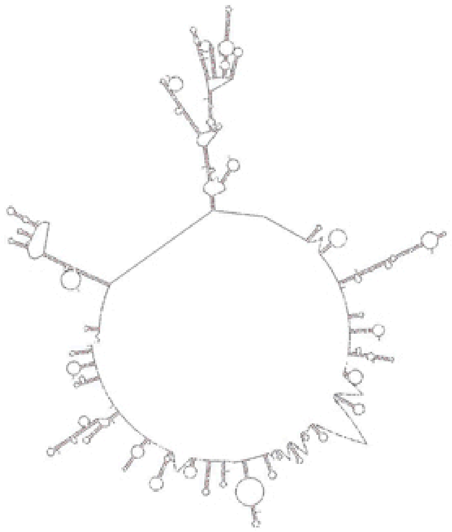

dG= -301.78 *TaeSat21-1590*

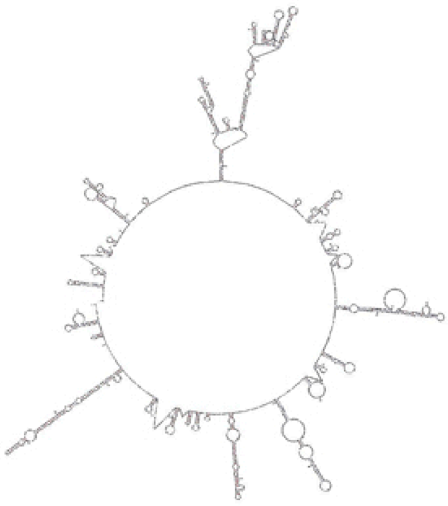

dG= -170.87 *TaeSat14-1463*

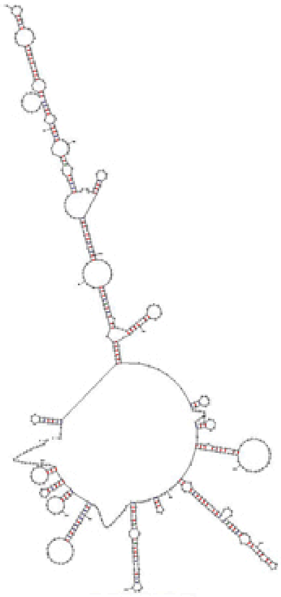

dG= -97.28 *TaeSat01-584*

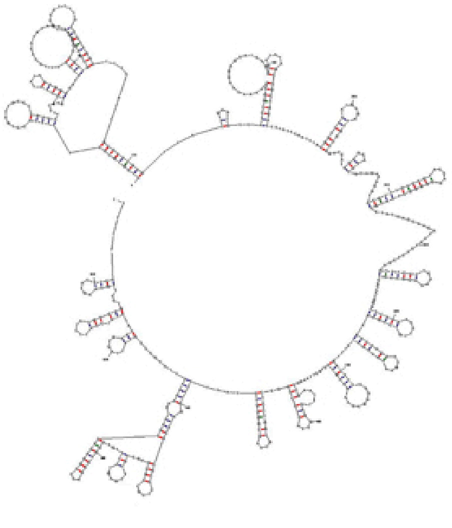

dG= -60.15 *TaeSat16-567*

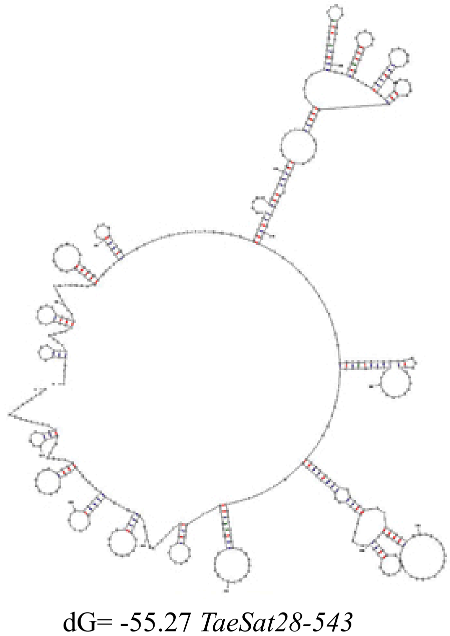

dG= -55.27 *TaeSat28-543*

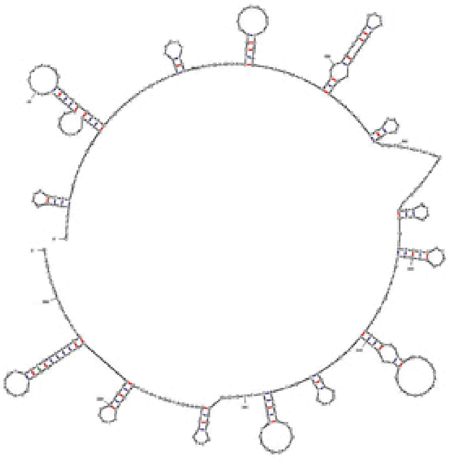

dG= -34.59 *TaeSat12-369*

Supplementary Figure S4. Alignment of the sequences belonging to superfamily 1 (SF-1): TaeSat04-337, TaeSat07-343 and TaeSat10-206. The duplicated AAATGATGAT sequence is tagged in yellow.

```
TaeSat04-337   ACGAGTTTCCGTATGAAACCCTGATACTTTGAAAGAGATTGTCCGTTTTG
TaeSat07-343   ATGAGTTCTCGTCCGAAACCCTGATACTCCGAAAGAGATTGTCCAGTTTG
TaeSat10-206   ACGAGTTTCCGTATGAAATCCTGATACTTTGAAAGAGATTGTCCGTTTTG
               * *****  ***  **** *********  **************  ****

TaeSat04-337   TACACGAAGTGCATCCAGTTTTTGCCGTAACCCTCTCTACTTTCTTGCAC
TaeSat07-343   TACACGAGGTGCGTCCAGTTTTCGCCGTGACCCTCTCTACTCTTTTGCAC
TaeSat10-206   TACACGAAGTGCATCCAGTTTTTGCCGTAACCCTCTCAACTTTCTTGCAC
               ******* **** ********* ***** ******** *** * ******

TaeSat04-337   ATGCTATGTGGGTGAAATGATGATACCATGCCAACTTTCAACCTTTTCAG
TaeSat07-343   ATGCTATGCGGGTGAAATGATGATACCATGCCAAGTTCCAACATTTTCAG
TaeSat10-206   ATGCTATGTGGATGAAATGATGAT--------------------------
               ******** ** ************

TaeSat04-337   AGTTCA-TTTGAAATGCTTTTCAATTTTAGGGTCTTATAGCTC---AAAA
TaeSat07-343   AGTTCATTTTGTAGTGATTTTCAATTTCACGGTCATTTAGCTCTCTAAAC
TaeSat10-206   --------------------------------------------------


TaeSat04-337   TAATTAGTAAATGCATGAAAAATAACAAATGAAGTCAGAAAGGATTGAAA
TaeSat07-343   AAATCGGTAAATGACTGAAAAACAGCAAATGATGTCAGAACGTGTTGGAA
TaeSat10-206   --------------------------------------------------


TaeSat04-337   AATGATGATGTGGCTTTGAATGGTGCATTTTGAACACACAAAAAGTCAGG
TaeSat07-343   ATTGATGACGTCGCTTTGAATGATGCATACTGAACGCAAAAATAGGCTGG
TaeSat10-206   ---------GTGGCTTTGAATGGTGCATTTTGAACACACAAAAAGTCAGG
                        ** ********** *****  ***** ** *** ** * **

TaeSat04-337   AGTTCAAATAAGTTTAAAAA--AATGAAATCCCTTTGTAACAG
TaeSat07-343   AGTTCAAATAAGTTTAAAAAACATTGAAGTGCCCGTGTAACAG
TaeSat10-206   AGTTCAAATAAGTTTTAAAA--AATGAAATCCCTTTGTAACAG
               *************** ****  * **** * **  ********
```

Supplementary Figure S5. Alignment of the sequences belonging to superfamily 2 (SF-2): TaeSat05-500 and TaeSat11-506.

```
TaeSat05-500    107 TTCAGGGTTCGTTTGGCCTTTTTAT-ACACT-AATTGAGTTTCCTAGGCATTTAATGTCC 164
TaeSat11-506    109 TTCAGGGGTCATTTGACCTTTTAAAGACATTTAAGTGATTTTC-TAACCATTTAATGACC 167
                    ******* ** **** ****** *  *** * ** *** **** **  ********* **

TaeSat05-500    165 ATAATTCAAATCTGAACTACAAATACATGCTCCAGTTCACCAAAATGGCTAGAAAAAT-- 222
TaeSat11-506    168 GTAATTCAAATTTGAACTACATCTACATGCAACGCCTAACCATAACGGTTTGAAAAATCA 227
                     ********** *********  *******  *   * **** ** ** * *******

TaeSat05-500    223 TATACATGTGTCCTTGGGTGCATGTTTAGGTCCCATG-CCAG-GAATGGGAACGAA-TTA 279
TaeSat11-506    228 TATTTTTGTGTACTTGTGTGCGAGTTAA--TTCCATGTGCAGTAAATTAGAAGGAATTTT 285
                    ***   ***** **** ****  *** *  * *****  ***  ***  *** *** **

TaeSat05-500    280 CAACCGTACCGGTGTCCTGGCTCGTCCTCAAACATTTCGAATC-TCGGTTTTTAAGTCCC 338
TaeSat11-506    286 CAAACATATTGGTCTCAAGGCATGGGCACATGCATGGAGTGCCATGGCATTTTAATT--C 343
                    *** * **  *** **  ***  *  * **  ***   *   * * *  ****** *  *

TaeSat05-500    339 CATAAATCCTAAACTCACCAG-AAAGTCATCAAAGGTGGCATGGTGTCACGTCATGGCAC 397
TaeSat11-506    344 CAAAAATTAAAAAATTATCAGAAAAAACATGAAACCTTCCTTGATGTAATGTCATGCCAC 403
                    ** ****   *** * * *** ***  *** ***  *  * ** *** * ****** ***

TaeSat05-500    398 ATATATGTCGTGGTAAAAACATTGCCA-ATTTGGGCCAAGCTTTATTACAAACCTCTTAC 456
TaeSat11-506    404 CAAGATGATGTGGTAAAAA-ATTGGCATGCTTGATGAAAGTTTGGACACACACCCCTCAC 462
                      * ***  ********** **** **   ***    *** **    *** *** ** **

TaeSat05-500    457 AAACCGGAGCCTGTCGCAAGAACCCTCGTGGTTTCGATAGGGAA 500
TaeSat11-506    463 AAACCGGAGCAACTCACTAGAAGGTTCGTGGTTCCGAGAGGGAA 506
                    **********   ** * ****   ******** *** ******
```

Supplementary Figure S6. Alignment of the sequences belonging to superfamily 3 (SF-3): TaeSat08-663 and TaeSat19-653.

```
TaeSat19-653     166 AGACCACCTTCGGCCGATTCAGCTGGAACCGGCTGGAATTTGAACTGCGGGTCCTCCATA  225
TaeSat08-663     180 AGACAAATTTCCGCCGATTCAGTTGGAAGCGGGTCAAATTTGAACTGCAGCTGCCTCATA  239
                     **** *  *** ********** ***** *** *  ************ * * *  ****

TaeSat19-653     226 GCTTGCCCGTTATTTTTGCTAAAAATGCTTTTTAGCTTCACAGGTAGGCATTTCATCACA  285
TaeSat08-663     240 GTTTGCTCTTTATTTTTTCCAAAAATCATTTCTAGGTACATAAGTATCTATTTAATCAGA  299
                     * **** * ******** * ******  *** *** * ** * ***   **** **** *

TaeSat19-653     286 GAATCGACAACAGATTGGCACGGCTCAAACCCTAGCCACGGACGGCC--GCCGCGACGAA  343
TaeSat08-663     300 GAAACACCAAAAAAATTCCAAGATTCAACCACTAGCTAGGAACGGTCATGCC-CGCCGTT  358
                     *** *  *** * * *  ** *  **** * ***** * * **** *  *** ** **

TaeSat19-653     344 ATCGGCCGGTTTTTGAAAACACCATAAAAAATTCAAAAAAA----------TGGGAGACC  393
TaeSat08-663     359 TT-GACCGCATTTTGAAACGGGCATAAAAAATTCAAAAAAAATCAAAAAATTGGAAAACC  417
                      * * ***  ********    *******************          *** * ***

TaeSat19-653     394 TCCGCGTCACATCATCAAATGTGGCCTACCAACTAGCAAAAATACTAAACTTGCAATACG  453
TaeSat08-663     418 TTCGCATTGTGTCATTATATGTGACCAAGTTTCCAGGAAAAATAATAAACTTGTAATACG  477
                     * *** *    **** * ***** ** *    * ** ******* ******** ******

TaeSat19-653     454 GATGTTTTCTTGAAAAAGTGTTCTCAAAAACGACCTACCATGAACGAAGATTCATGGCTT  513
TaeSat08-663     478 GCAATTATTTTAAAAAAGTGTTCTCAGAAATGAGCTATCATGTGTGAAGATTCATGGCTT  537
                     *   ** * ** ************** *** ** *** ****   ***************

TaeSat19-653     514 TCAAGCCAAACTAGCAATGATATGGCCGCATCCGTTGAATAGTTTCTGATAATATGCCCA  573
TaeSat08-663     538 TCAAGCCAAATGATCAATCTTATGGCCACATTCATGGCATAGTTTGTTCAAATGATCTCA  597
                     **********  * ****  ******* *** * * * ******* *   ***   * **

TaeSat19-653     574 AATTTGGCGCATGCCTCCGTCTTGTGATGGCAAACAATGTTGCCAAAGCGAGGTTCCAAC  633
TaeSat08-663     598 TATTGTGCACAAGGGTGCATATTGGAATGGCAAACAATGTTGCCTAAGGAAGTTTTCATT  657
                      ***  ** ** *  * * * ***  ****************** ***  ** ** **

TaeSat19-653     634 TTGTTT  639
TaeSat08-663     658 TTCTTT  663
                     ** ***
```

Supplementary Figure S7. Alignment of the sequences belonging to superfamily 4 (SF-4): TaeSat09-335 and TaeSat20-322.

```
TaeSat09-335 3    CTCCGGGGTGTGGCCCCCCTC-ACGGACGGCGCCGCCGCCGGCACCTGGAGGGGGGAAA-  60
TaeSat20-322 1    CTCCGTGGTGTGGCCCCCCTCCACGGAAGGCAGAGCAGCCGTCACTTAGAGGGGGGATGG  60
                  ***** *************** ***** ***   ** **** *** * *********

TaeSat09-335 61   --CGGACGCGTGGTAACTACACGGAGGGGATCTTGCTGTGGGTCTGG---AGCGCGTGTT  115
TaeSat20-322 61   TGCGGGTGCGGTGGAAC----CGGAGGGAATCTAGTGTTGGGTTGGGTCCAGACCGTGTT  116
                    ***  ***  * ***    ******* **** *   *****  **   **  ******

TaeSat09-335 116  GCTCCAAAGTGTTCCTATGGGCTGACCTAACACAACCGGGTGGA-GAGGTTTTTACTGGT  174
TaeSat20-322 117  CGGGGA---TGTTGCTATGGGCTGACCTATCGCAACCAGGTGGAAGAG---TCCACTGGT  170
                       *   **** *************** * ***** ****** ***   *  ******

TaeSat09-335 175  CAAAGCCCATCCGTGCAAAGTCAAAGGGCTAGATCCCGTGGTCAAACGCTACAGGGTTAG  234
TaeSat20-322 171  CAAAGCCCGTCCGGCAAAAGTCAAAGGGCTAGATCTCCTGGTCAA---CT--GGGATTCG  225
                  ******** ****   ******************* * *******   **   ** ** *

TaeSat09-335 235  GCGGGACGGGGGCCTCGGGGGGCTAG-AATGCCACCGGAGCGTCGGTAGGCGCCCTCATA  293
TaeSat20-322 226  G------GGTGGCCTTCGGGGTGTAGCTATGCCACCGAAACATC-GCACGGGTCCTGATG  278
                  *      ** *****  ****  ***  ********* * * ** * * * * *** **

TaeSat09-335 294  CATGCGGGGTGGTGTTGTGGCATG  317
TaeSat20-322 279  CATGTGTGAAAGTGTTCTGGCATG  302
                  **** * *   ***** *******
```

Supplementary Figure S8. Alignment of the sequences belonging to superfamily 6 (SF-6): TaeSat22-320, TaeSat23-319, TaeSat25-318 and TaeSat29-319.

```
                  ** ** ***** **    *******  *****    **** *********
TaeSat25-318  116 GGCCCCCACGCAAGGTTGGAACGAAAACGGACACAAAACGGACGTTGCGT 165
TaeSat23-319  155 GGACCGCACGCCAGAGATGAACGAAAACGGACTACGAACGCACGTTGCGT 204
TaeSat22-320  148 GGGCCCCACGCAAGATTTGAACGAATTCGGACACCAAACGCACGTTGCGT 197
TaeSat29-319  213                                     AACGCAAGTTGCGT 226
                                                      **** * *******

                  ****  *** ********* *     **        **** * **  ***
TaeSat25-318  166 CACGTCCGGGCAGTGTTTTTGCGATTTTGGACCTGGAAAACCCTAGAAAA 215
TaeSat23-319  205 CACGTGCGGGCAGTGTTTTAGGGGTTTTCGGTGCGAAAAATCATAGAAAA 254
TaeSat22-320  198 CACGCCCGGTCAGTGTTTTCGGTGCGTTTCACGGAGAAAACCATAAGAAA 247
TaeSat29-319  227 CACGTCGGGAGACCTTTTCTGGGTATTTCCACGGAGAAAAACGTAGAAAA 275
                  ****   **  *   ***  *     **        **** * **  ***

                  * * *   * *******
TaeSat25-318  216 TGCGTGGAGCGTCGGAA 232
TaeSat23-319  255 TCCGTACAGCGTCGGAA 271
TaeSat22-320  248 TTCATACGGTGTCGGAA 264
TaeSat29-319  276 TGCATCGACTGTCGAAA 292
                  * * *     **** **
```

Supplementary Figure S9. Alignment by pairs of the sequences belonging to superfamily 6 (SF-6): TaeSat22-320, TaeSat23-319, TaeSat25-318 and TaeSat29-319.

```
TaeSat29-319 132  CGAAACGCACCCAACTTTTGCGTGCGCGTGTGAGTGACCCCCACCGGCTCGCACGCCTAT  191
TaeSat23-319 110  CGGAATGCACCCAACTTTTGGCAGCGCGTGTGGGGCCCCGCCCGGGGACCGCACGCCAGA  169
                  ** ** **************   ********* *   ** **   **  ********

TaeSat29-319 192  TTTCGACGGAATCGGAGTACGAACGCAAGTTGCGTCACGT-CGGGAGACCTTTTCTGGGT  250
TaeSat23-319 170  GATGAACGAAAACGGACTACGAACGCACGTTGCGTCACGTGCGGGCAGTGTTTTAGGGGT  229
                    *  *** ** **** ********** ************ ****     ****  ****

TaeSat29-319 251  ATTTCCACGGAGAAAAACGTAGAAAATGCATCGACTGTCGAAACGCACCCCAATTTTGCA  310
TaeSat23-319 230  -TTTCGGTGCGAAAAATCATAGAAAATCCGTACAGCGTCGGAACGC-GACAAATTTT---  284
                   ****   *   **** * ******** * *  *  **** *****   * ******

TaeSat29-319 311  GGCGTGC  317
TaeSat23-319 285  GGCGTGC  291
                  *******

---------------------------------------------------------------------------------

TaeSat29-319 126  CATGGACGAAACG-CACCCAACTTTTGCGTGCGCGTGTGAGTGACCCCCACCGGCTCGCA  184
TaeSat22-320 97   CATGCATGAAACGTCACCCAA-TTTTGGGAGGCAGTGGGCATGCCCATCTTAGGGCCCCA  155
                  **** * ****** ******* ***** * *   *** *  ** **  *   **  * **

TaeSat29-319 185  CGCCTATTTTCGACGGAATCGGAGTACGAACGCAAGTTGCGTCACGTCGGGAGA-CCTTT  243
TaeSat22-320 156  CGCAAGATTTGAACGAATTCGGACACCAAACGCACGTTGCGTCACGCCCGGTCAGTGTTT  215
                  ***    ***  *** * *****   * ****** *********** * **  *   ***

TaeSat29-319 244  TCTGGGTATTTCCACGGAGAAAAACGTAGAAAATGCAT  281
TaeSat22-320 216  TCGGTGCGTTT-CACGGAGAAAACCATAAGAAATTCAT  252
                  ** * *  *** *********** * **  **** ***

---------------------------------------------------------------------------------

TaeSat29-319 213  AACGCAAGTTGCGTCACGTCGGGAGACCTTTTCTGGGTATTTCCACGGAGAAAAACGTAG  272
TaeSat25-318 152  AACGGACGTTGCGTCACGTCCGGGCAGTGTTTTTGCGATTTTGGACCTGGAAAACCCTAG  211
                  **** * ************* **  *   *** ** *  ***  **   ***** * ***

TaeSat29-319 273  AAAATGCATCGACTGTCGAAA  293
TaeSat25-318 212  AAAATGCGTGGAGCGTCGGAA  232
                  ******* * **  **** **
---------------------------------------------------------------------------------
```

```
TaeSat23-319 155  GGACCGCACGCCAGAGATGAACGAAAACGGACTACGAACGCACGTTGCGTCACGTGCGGG  214
TaeSat22-320 148  GGGCCCCACGCAAGATTTGAACGAATTCGGACACCAAACGCACGTTGCGTCACGCCCGGT  207
                  ** ** ***** ***  ********  *****  * ******************  ***

TaeSat23-319 215  CAGTGTTTTAGG-GGTTTTCGGTGCGAAAAATCATAGAAAATCCGTACAGCGTCGGAA  271
TaeSat22-320 208  CAGTGTTTTCGGTGCGTTTCACGGAG-AAAACCATAAGAAATTCATACGGTGTCGGAA  264
                  ********* ** *  ****   * * **** ****  **** * *** * *******

--------------------------------------------------------------------------------

TaeSat23-319 155  GGACCGCACGCCAGAGATGAACGAAAACGGACTACGAACGCACGTTGCGTCACGTGCGGG  214
TaeSat25-318 116  GGCCCCCACGCAAGGTTGGAACGAAAACGGACACAAAACGGACGTTGCGTCACGTCCGGG  175
                  ** ** ***** **    **************    **** ************** ****

TaeSat23-319 215  CAGTGTTTTAGGGGTTTTCGGTGCGAAAAATCATAGAAAATCCGTACAGCGTCGGAA  271
TaeSat25-318 176  CAGTGTTTTTGCGATTTTGGACCTGGAAAACCCTAGAAAATGCGTGGAGCGTCGGAA  232
                  ********* * * **** *    * **** * ******** ***  **********

--------------------------------------------------------------------------------

TaeSat22-320 61   CCGATCAGT-GCCGGTTGCATGGGTGGTACGAGGTGGCATGCATGAAACGTCACCCAATT  119
TaeSat25-318 28   CCGACAAGTAGCTGGTTGCACTGGGGCTACGTGATCGCATGCATGCAACTGCACCAAAGT  87
                  ****  *** ** *******  ** * **** * * ********* ***  **** ** *

TaeSat22-320 120  TTGGGAGGCAGTGGGCATGCCCATCTTAGGGCCCCACGCAAGATTTGAACGAATTCGGAC  179
TaeSat25-318 88   GTGCGTACATGTGGGCACGGCCAACGTAGGCCCCCACGCAAGGTTGGAACGAAAACGGAC  147
                   ** *     ******* * *** * **** *********** ** *******  *****

TaeSat22-320 180  ACCAAACGCACGTTGCGTCACGCCCGGTCAGTGTTTTCGGTGCGTTTC---ACGGAGAAA  236
TaeSat25-318 148  ACAAAACGGACGTTGCGTCACGTCCGGGCAGTGTTTT---TGCGATTTTGGACCTGGAAA  204
                  ** ***** ************* **** *********   **** **    **   ****

TaeSat22-320 237  ACCATAAGAAATTCATACGGTGTCGGAAAATTATGAAAACTTGCACGCATGATGGCCCTG  296
TaeSat25-318 205  ACCCTAGAAAATGCGTGGAGCGTCGGAAAAATACGCGAGTTGGCGTGGGTGCTGTGGATG  264
                  *** **  **** * *   * ********* ** *  *  * **  *  ** **    **

TaeSat22-320 297  GTGATGAGTGCGTGTGGAAAAAGT  320
TaeSat25-318 265  GTGATGGCAACGTGTGGAAAAAGT  288
                  ******    **************
```

Supplementary Figure S10. Idiograms of chromosomes of T. aestivum cv. Chinese Spring bread wheat representing satellites DNA location identified in this work by fluorescent in situ hybridization (FISH). (a) satDNAs with terminal location (telomeric and subtelomeric); (b) satDNAs with multiple locations (terminal, centromeric and interstitial), and (c) satDNAs with (peri)centromeric location.

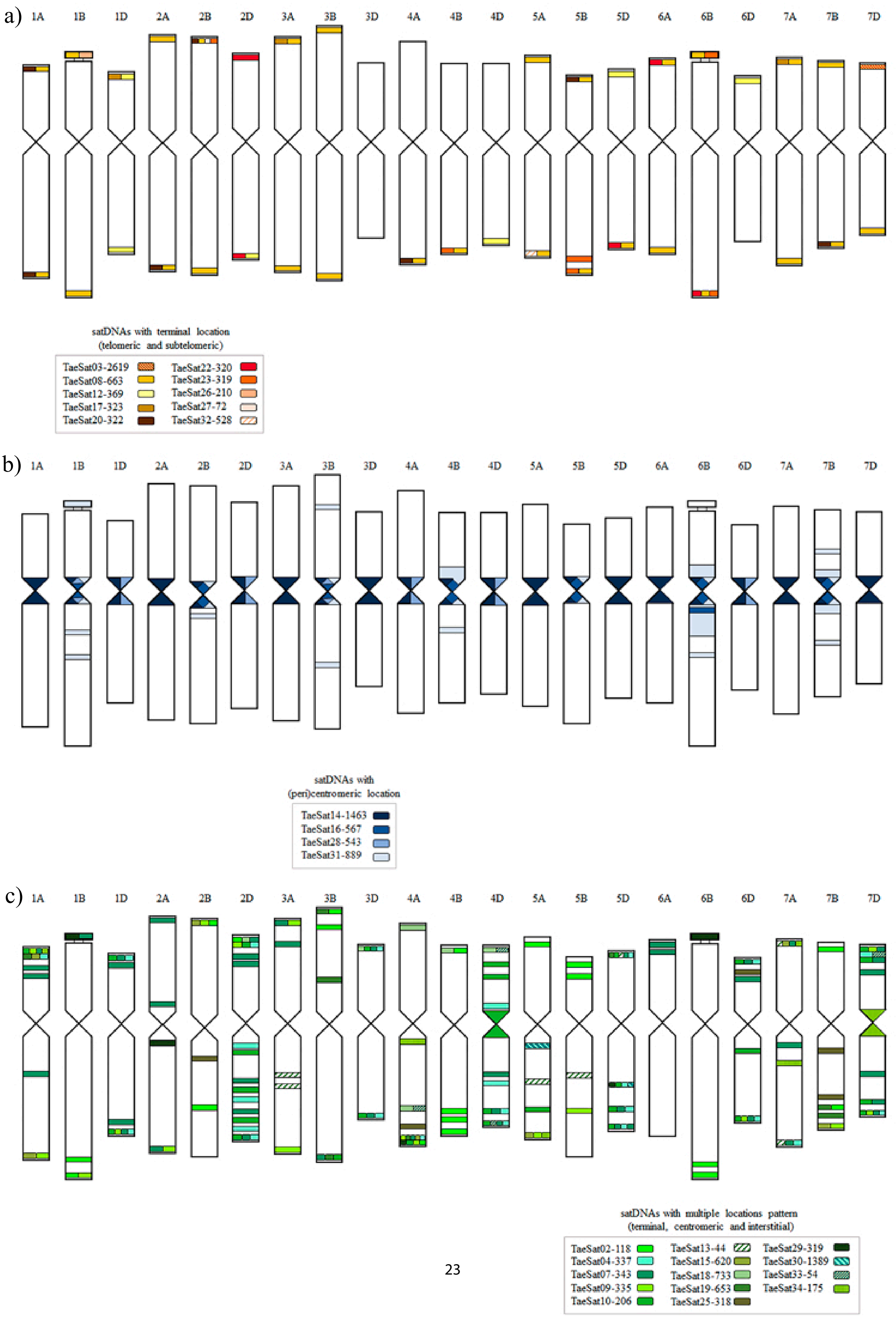

Supplement: Supplementary file 1 — Supplementary file1 (PDF 21048 KB) [file 11103_2023_1404_MOESM1_ESM.pdf]
